# Quantitative Indices for Drought Tolerance in Rice: Leveraging Genetic Resources for Climate‐Resilient Breeding

**DOI:** 10.1002/pei3.70145

**Published:** 2026-05-14

**Authors:** Alireza Tarang, Alireza Haghighi Hasanalideh

**Affiliations:** ^1^ Rice Research Institute of Iran, Agricultural Research, Education and Extension Organization (AREEO) Rasht Iran

**Keywords:** drought tolerance, GGE biplot, multivariate analysis, stress indices

## Abstract

Climate change‐induced drought is a major threat to global rice production, underscoring the urgent need for drought‐resilient cultivars. This study aimed to identify drought‐tolerant, high‐yielding rice genotypes using quantitative yield‐based indices and multivariate analyses. Eighty‐two diverse genotypes were evaluated over three seasons (2020–2022) under two moisture regimes (normal irrigation and terminal drought stress), constituting six environments in total. Data were analyzed using ANOVA, correlation, GGE biplot, and principal component analysis. Results revealed significant genotype, environment, and genotype‐by‐environment interaction effects, highlighting substantial genetic diversity. Mean grain yield under normal irrigation was 4428 kg/ha, which declined to 2676 kg/ha under terminal drought stress, a reduction of 39.58%, primarily due to a 26.85% decrease in filled grains per panicle. Genotypes M1, Sela‐Zodras, and Shiroudi were consistently identified as superior, demonstrating high yield under both normal and stress conditions, coupled with outstanding STI, GMP, and MP values. Correlation and PCA analyses confirmed high collinearity among STI, GMP, MP, and Yield Index, establishing their reliability for simultaneous selection of high yield potential and drought tolerance. GGE biplot analysis further validated M1 as an ideal genotype with superior yield and stability. We recommend STI, GMP, and MP as the most effective indices for drought tolerance screening. The elite genotypes M1, Sela‐Zodras, and Shiroudi represent valuable genetic resources. They are recommended for further multi‐location testing under terminal drought conditions and, pending validation, for potential direct cultivation in drought‐prone areas or as donor parents in breeding programs to enhance climate resilience in rice.

## 1 Introduction

Rice ( *Oryza sativa* L.) stands as a historically vital crop that plays an important role in food security for over half of the world's population (Mas‐Ud et al. 2022). But this vital food source is under threat. Climate change is driving more frequent and intense drought periods, pushing agricultural systems to their breaking point (Kandel et al. 2022). The consequences of drought stress are unequivocal, capable of wiping out as much as half of the potential yield (Kumar et al. 2014). This directly translates into severe economic losses and intensified food insecurity. So, cultivating drought‐resilient rice varieties has transcended the realm of academic interest to become an urgent global priority (Abd El‐Aty et al. 2022). Drought stress severely curtails rice productivity by triggering detrimental morphological and physiological changes. It inhibits key processes like germination, tillering, and photosynthesis, ultimately stunting growth and reducing biomass. The plant's reproductive phase is especially sensitive, where water deficit leads to substantial yield loss through impaired grain development (Bhandari et al. 2023).

Modern breeding programs, while successful in enhancing yield, have largely relied on a narrow genetic base, potentially limiting the availability of adaptive traits necessary for stress resilience. This genetic purification of modern agroecosystems has provoked severe genetic erosion, heightening vulnerability to climatic volatility. Therefore, the systematic evaluation of ex‐situ germplasm collections is not merely an academic pursuit but a vital risk mitigation strategy—effectively creating a genetic insurance policy against future abiotic stress scenarios (McCouch et al. 2020). This genetic bottleneck underscores the critical need to explore untapped genetic resources, particularly traditional landraces and wild relatives, which harbor valuable alleles conferring drought adaptation evolved through natural selection in marginal environments (Kumar et al. 2014). Consequently, the development of efficient methods for quantitatively assessing drought tolerance using yield‐based indices has gained attention as a fundamental approach in breeding programs (Sabouri et al. 2022). Valuing these genetic resources represents a strategic mining operation for adaptive genes, and phenotypic and genotypic screening of diverse germplasm accessions has revealed superior traits associated with critical drought stress mechanisms (McCouch et al. 2020).

Yield‐based drought indices represent powerful tools for assessing drought tolerance in rice. Among various indices, Stress Tolerance Index (STI), Geometric Mean Productivity (GMP), and Mean Productivity (MP) are recognized as reliable criteria due to their strong correlation with yield across different conditions. However, challenges such as environmental variability, genotype × environment interactions, and the need for standardization of evaluation methods persist. The theoretical foundations of quantitative drought tolerance indices trace back to the research of Fischer and Maurer (1978), who first introduced the Stress Susceptibility Index (SSI). This index is calculated based on the relative yield reduction under stress conditions compared to optimal conditions. Subsequently, Fernandez (1992) proposed the Stress Tolerance Index (STI), which enables the identification of genotypes exhibiting high performance under both stress and non‐stress conditions. Additionally, Rosielle and Hamblin (1981) defined the Tolerance Index (TOL) as the absolute difference between yield under optimal and stress conditions, while Gavuzzi et al. (1997) developed the Yield Index (YI) to assess the relative yield of genotypes under stress.

Recent research consistently validates this toolkit. Studies by Kandel et al. (2022) and Hussain et al. (2021) have confirmed that indices like STI, GMP, and MP show a strong positive correlation with yield under stress and are therefore reliable criteria for selection. Nagaraju et al. (2023) further refined this, demonstrating that certain indices are particularly effective at identifying robust, stable lines, while others excel at screening out susceptible lines. The heritability of key traits like tiller number and grain weight, as shown by (Abbas et al. 2025), confirms that selection for these drought‐linked characteristics is both feasible and effective.

To identify superior, stable genotypes and the most reliable drought indices, this study evaluated 82 rice genotypes under normal and drought stress conditions using a suite of yield‐based indices.

## 2 Materials and Methods

This experiment was conducted at the Rice Research Institute of Iran (RRII, Rasht; 37°12′ N, 49°38′ E) over three consecutive cropping seasons (2020–2022). In this study, a diverse panel of 82 rice genotypes with varied genetic and geographical backgrounds was utilized.

### 2.1 Plant Material

The genotypes were sourced from multiple countries across the region, including Iran (19 genotypes), Iraq (18 genotypes), Turkey, Azerbaijan, Afghanistan, Uzbekistan, Kazakhstan, Kyrgyzstan, as well as the International Rice Research Institute (IRRI). The collection comprised three main categories: local cultivars, breeding lines, and released cultivars, representing a broad spectrum of the genetic diversity available in regional gene banks (Table 1). Employing this wide‐ranging panel enabled a comprehensive evaluation of stress tolerance‐related traits and the identification of superior genotypes with potential for use in future breeding programs.

**TABLE 1:** List of 82 rice genotypes evaluated in the study, including genotype code, name, origin, and type (local cultivar, breeding line, or released cultivar).

Code	Genotype	Origin	Type of genotype	Code	Genotype	Origin	Type of genotype
1	A16	Iraq	Breeding line	42	Pasali	Turkey	Cultivar
2	Ak‐ypyk	Kyrgyzstan	Local cultivar	43	QazNIIR‐7	Kazakhstan	Local cultivar
3	Amber albalaka	Iraq	Local cultivar	44	Sela‐Zodras	Afghanistan	Local cultivar
4	Anbar33	Iraq	Local cultivar	45	Shalawangi 1	Afghanistan	Local cultivar
5	Atai‐1	Afghanistan	Local cultivar	46	Shalawangi 2	Afghanistan	Local cultivar
6	Barnamaj4	Uzbekistan	Local cultivar	47	Siyavar Hasimi	Azerbaijan	Local cultivar
7	BT7	Iraq	Breeding line	48	Sumer	Iraq	Local cultivar
8	C10	Iraq	Breeding line	49	Syl Sulu	Kazakhstan	Local cultivar
9	Cakmak	Turkey	Cultivar	50	T85	Iraq	Breeding line
10	D3	Iraq	Breeding line	51	Tantana	Azerbaijan	Local cultivar
11	Dijla	Iraq	Local cultivar	52	Tarona	Azerbaijan	Local cultivar
12	Dollar	IRRI	Cultivar	53	TosyaGunesi	Turkey	Cultivar
13	Furat1	Iraq	Local cultivar	54	V20‐48(awn)	Kazakhstan	Breeding line
14	Ghadeer	Iraq	Local cultivar	55	V20‐53‐2‐2	Kazakhstan	Breeding line
15	Halibey	Turkey	Cultivar	56	V20‐8‐2	Kazakhstan	Breeding line
16	Hikkan Hashimi	Uzbekistan	Local cultivar	57	Xazaz Hazar	Uzbekistan	Local cultivar
17	HT1	Iraq	Breeding line	58	Yasmine	Azerbaijan	Local cultivar
18	Iba	Iraq	Local cultivar	59	Ahlami Tarom	Iran	Local cultivar
19	IR28	IRRI	Cultivar	60	Abjiboji	Iran	Cultivar
20	IR30	IRRI	Cultivar	61	Bojar	Iran	Cultivar
21	IR36	IRRI	Cultivar	62	Binam	Iran	Local cultivar
22	IR50	IRRI	Cultivar	63	Champa Boodar	Iran	Local cultivar
23	IR58	IRRI	Cultivar	64	Hasansaraei	Iran	Local cultivar
24	IR60	IRRI	Cultivar	65	Hasani	Iran	Local cultivar
25	IR64	IRRI	Cultivar	66	Khazar	Iran	Cultivar
26	Iskander	Azerbaijan	Local cultivar	67	Domzard	Iran	Local cultivar
27	Jalal Abad	Afghanistan	Local cultivar	68	Domsefid	Iran	Local cultivar
28	Kapa	Kyrgyzstan	Local cultivar	69	Domsiah	Iran	Local cultivar
29	Kawther	Iraq	Local cultivar	70	Sahel	Iran	Cultivar
30	Labypma	Azerbaijan	Local cultivar	71	Salari	Iran	Cultivar
31	LT2	Iraq	Breeding line	72	SangTarom	Iran	Local cultivar
32	M1	Iraq	Breeding line	73	Shahpasand	Iran	Local cultivar
33	Manyas Yildizi	Turkey	Cultivar	74	Shiroudi	Iran	Cultivar
34	Marjan	Kazakhstan	Local cultivar	75	Saleh	Iran	Cultivar
35	Mis‐2013	Turkey	Cultivar	76	Alikazemi	Iran	Local cultivar
36	Mishkab1	Iraq	Breeding line	77	Anbarboo	Iran	Local cultivar
37	Mishkab2	Iraq	Breeding line	78	Gharib	Iran	Local cultivar
38	Mustakillik	Azerbaijan	Local cultivar	79	Kadous	Iran	Cultivar
39	Okean	Uzbekistan	Local cultivar	80	Gilaneh	Iran	Cultivar
40	osmancik‐97	Turkey	Cultivar	81	Mohammadi	Iran	Local cultivar
41	Avangard	Uzbekistan	Local cultivar	82	Hashemi	Iran	Local cultivar

### 2.2 Field Experiment

The field experiment was conducted using a randomized complete block design (RCBD) with three replications to account for potential field heterogeneity. The reliability of yield estimates (expressed in kg/ha) depends on uniform plot management and precise area measurement. To further control variability, uniform pre‐planting soil preparation was conducted. Soil samples were collected prior to planting to confirm uniformity in pH, organic matter, and nutrient availability across blocks. Although the RCBD effectively controls for gradient‐based heterogeneity, the presence of significant genotype × environment interaction observed in the ANOVA confirms that multi‐environment testing remains essential for evaluating stability and adaptability.

The genotypes were evaluated under two moisture regimes: (1) normal irrigation, and (2) terminal drought stress. The experiment was laid out in plots measuring 2 × 2 m, with a planting density of 16 plants per square meter. Drainage trenches were constructed around the field to prevent water ingress and ensure rapid drainage. Fertilizers were applied at rates of 200 kg N/ha (as urea), 100 kg P_2_O_5_/ha (as triple superphosphate), and 100 kg K_2_O/ha (as potassium sulfate). Weed control was achieved by applying the herbicide Purtilar at 1.5 L/ha 7 days after transplanting, supplemented by manual weeding throughout the growth stages.

In the normal field, plots were maintained flooded until 1 week before harvest. In the stress environment, irrigation was permanently discontinued at the maximum tillering stage (~55–60 days after transplanting, corresponding to growth stage IT) and withheld until physiological maturity, imposing a terminal drought stress lasting approximately 40–45 days. Ten days after irrigation cessation, soil water potential was recorded every 5 days (Table S1). The meteorological data indicates significant climatic variability, particularly in the distribution and amount of monthly rainfall. Despite this variable background, the controlled irrigation withholding protocol applied as the main treatment successfully induced a strong, consistent, and predictable declining trend in soil moisture status. The results show that, regardless of initial precipitation conditions, withholding irrigation led to a rapid decrease in soil water potential from values near field capacity (approximately −0.8 kPa) to highly negative values well beyond the permanent wilting point (down to −1370 kPa) within 30 days (Table S2). This reproducible pattern of gradual and intensifying soil drying provided a standardized and uniform experimental platform for evaluating and comparing the physiological and morphological responses of rice genotypes to varying levels of water deficit stress across consecutive years. Indeed, this approach enabled the isolation of intrinsic drought tolerance traits from the effects of random climatic fluctuations, thereby significantly enhancing the comparability and robustness of the research findings.

### 2.3 Data Collection, Processing and Statistical Analysis

The following agronomic traits were recorded according to the Standard Evaluation System for Rice (SES 2014):

Plant height (cm):

Measured from the soil surface to the tip of the highest panicle at maturity.

Number of fertile tillers per plant:

Counted as the number of productive tillers bearing panicles at harvest.

Panicle length (cm):

Measured from the panicle base to the tip of the main panicle.

Flag leaf length (cm):

Length of the flag leaf measured from the ligule to the leaf tip.

Number of filled grains per panicle:

Total number of fully developed grains per panicle.

Grain yield (kg/ha):

Total grain weight per plot, adjusted to 14% moisture content and converted to kg per hectare.

1000‐grain weight (g):

Weight of 1000 randomly selected filled grains.

Kernel length (mm):

Length of 10 randomly selected debusked grains, averaged.

Analysis of variance (ANOVA) was performed using the following linear mixed model for the combined multi‐environment trial:

$$Y_{\mathrm{ijk}}=\mu +G_{i}+E_{j}+Y_{k}+{\left(G\times E\right)}_{\mathrm{ij}}+{\left(G\times Y\right)}_{\mathrm{ik}}+{\left(E\times Y\right)}_{\mathrm{jk}}+{\left(G\times E\times Y\right)}_{\mathrm{ijk}}+B_{l\left(\mathrm{jk}\right)}+\varepsilon_{\text{ijkl}}$$

where $Y_{\mathrm{ijk}}$ = observed trait value, μ = overall mean, $G_{i}$ = fixed effect of genotype *i*, $E_{j}$ = fixed effect of environment *j* normal vs. stress, $Y_{k}$ = fixed effect of year *k*, ${\left(G\times E\right)}_{\mathrm{ij}}$ = genotype × environment interaction, ${\left(G\times Y\right)}_{\mathrm{ik}}$ = genotype × year interaction, ${\left(E\times Y\right)}_{\mathrm{jk}}$ = environment × year interaction, ${\left(G\times E\times Y\right)}_{\mathrm{ijk}}$ = three‐way interaction, $B_{l\left(\mathrm{jk}\right)}$ = random effect of block *l* nested within environment and year, $\varepsilon_{\text{ijkl}}$ = residual error.

A suite of drought tolerance indices was calculated based on grain yield under non‐stress (Yn) and stress (Ys) conditions, using the formulae presented in Table 2.

**TABLE 2:** Formulas and references for the drought tolerance and susceptibility indices used to evaluate rice genotypes under normal and terminal drought stress conditions.

Drought tolerance indices	Equation	References
Stress Susceptibility Index (SSI)	$\left(1-\left(Y_{\mathrm{si}}/Y_{\mathrm{ni}}\right)\right)/\left(1-\left(\;{\bar{Y}}_{s}/{\bar{Y}}_{n}\right)\right)$	Fischer and Maurer (1978)
Tolerance index (TOL)	$Y_{\mathrm{ni}}-Y_{\mathrm{si}}$	Rosielle and Hamblin (1981)
Mean productivity (MP)	$\left(Y_{\mathrm{ni}}+Y_{\mathrm{si}}\right)/2$	(Rosielle and Hamblin 1981)
Geometric mean productivity (GMP)	$\sqrt{Y_{\mathrm{ni}}.Y_{\mathrm{si}}}$	Rosielle and Hamblin (1981)
Stress tolerance index (STI)	$\left(Y_{\mathrm{ni}}.Y_{\mathrm{si}}\right)/\left({\bar{Y}}_{n}^{2}\right)$	Fernandez (1992)
Yield Index (YI)	$Y_{\mathrm{si}}/{\bar{Y}}_{s}$	Gavuzzi et al. (1997)
Yield stability index (YSI)	$Y_{\mathrm{si}}/Y_{\mathrm{ni}}$	Bouslama and Schapaugh (1984)
Harmonic Mean (HM)	$\left(2.Y_{\mathrm{ni}}.Y_{\mathrm{si}}\right)/\left(Y_{\mathrm{ni}}+Y_{\mathrm{si}}\right)$	Fernandez (1992)
Relative drought index (RDI)	$\left(Y_{\mathrm{si}}/Y_{\mathrm{ni}}\right)/\left({\bar{Y}}_{s}/{\bar{Y}}_{n}\right)$	Fischer and Wood (1979)
Abiotic Tolerance Index (ATI)	$\left(\left(Y_{\mathrm{ni}}-Y_{\mathrm{si}}\right)/\left({\bar{Y}}_{n}/{\bar{Y}}_{s}\right)\right)\left(\sqrt{Y_{\mathrm{ni}}.Y_{\mathrm{si}}}\right)$	Moosavi et al. (2008)

*Note:* $Y_{\mathrm{ni}}$: yield of genotype i under non‐stress; $Y_{\mathrm{si}}$: yield of genotype i under stress; ${\bar{Y}}_{n}$: mean yield of all genotypes under non‐stress; ${\bar{Y}}_{s}$: mean yield of all genotypes under stress.

Analysis of variance (ANOVA) and principal component analysis (PCA) were performed using the SAS software. PCA biplots were generated with SPSS software. GGE biplot visualizations were constructed to assess genotype‐by‐environment interactions and identify superior genotypes using GenStat version 12.

## 3 Results

The combined analysis of variance revealed that the effect of genotype was significant for all traits, indicating substantial genetic diversity among the studied genotypes. The environmental effect was also highly significant for all traits, demonstrating that drought stress significantly influenced trait expression. A significant genotype × environment interaction was observed for most traits, revealing that the ranking of genotypes varied across different environments and underscoring the necessity for multi‐environment evaluations. Thus, the identification of genotypes exhibiting both high yield stability and broad adaptability is imperative. Furthermore, the genotype × year and environment × year interactions were significant for a majority of the traits, highlighting the variability of genotypes across different years and the fluctuating nature of environmental conditions over time (Table 3).

**TABLE 3:** Combined analysis of variance (ANOVA) for agronomic traits of 82 rice genotypes evaluated across 3 years and two moisture regimes (normal irrigation and terminal drought stress).

S.O.V.	df	Mean square							
		Height	Fertile tiller	Panicle length	Flag leaf length	Full filled grain per panicle	Yield	1000‐grain weight	Kernel length
Y	2	27.73^ns^	174.64**	0.46^ns^	176.13**	15629.31**	244084.00^ns^	42.05**	5.69**
En	1	77620**	1153.81**	13309.21**	25805.32**	368095.85**	1133395204.00**	4678.77**	13.53**
Y×En	2	20055.1**	36.70**	3194.79**	288.80**	14820.09**	8242872.00**	228.44**	5.22**
R(Y×En)	12	13.18	2.71	1.65	6.39	38.32	109036.00	0.99	0.01
G	81	7585.52**	92.41**	356.85**	507.21**	11894.36**	8118397.00**	595.53**	6.33**
Y×G	162	315.57**	17.57**	21.58**	82.42**	1089.70**	1297871.00**	10.85**	0.78**
En×G	81	44.79**	5.78**	11.56**	40.70**	799.81**	711687.00**	9.63**	1.13**
Y×En×G	162	44.55**	3.02**	8.67**	27.78**	320.99**	317813.00**	9.15**	0.77**
Error	972	7.39	1.07	5.34	3.35	23.52	130,414	0.65	0.01
CV		2.21	8	8.84	6.12	4.76	10.17	3.09	1.38

*Note:* ns, * and **, no significance, significance at *p* ≤ 0.05, and *p* ≤ 0.01, respectively.

Abbreviations: CV, coefficient of variation; df, degrees of freedom; En, environment (normal vs stress); G, genotype; R, replicate; Y, year.

Mean evaluation results (Tables S3 and S4) identified Genotype M1 (G32), with a grain yield of 4830.6 kg/ha, as the top‐yielding genotype. Superior performance for specific yield components was demonstrated by Genotype Manyas Yildizi (G33) (175.89 filled grains per panicle), Genotype Shalawangi 2 (G46) (1000‐grain weight of 37.28 g), and Genotype IR28 (G19) (panicle length of 34.53 cm). The maximum and minimum kernel lengths were observed in IR58 (G23) (7.86 mm) and V20‐8‐2 (G56) (5.54 mm), respectively. The considerable disparity between the minimum and maximum values for key traits, such as the number of fertile tillers (7.44–17.13) and the number of full filled grains per panicle (44.17–175.89), signifies a significant potential for selection to enhance yield within this germplasm (Tables S3 and S4).

The most decline was observed in grain yield, which decreased from 4428 kg/ha under normal conditions to 2676 kg/ha under stress, representing a reduction of 39.58%. This substantial yield loss can be largely attributed to a 26.85% decrease in fully filled grains per panicle, which dropped from 117.68 to 86.09 grains (Table S4).

Significant reductions were also recorded in other yield components and morphological characteristics. Panicle length (20.71%), flag leaf length (24.51%), number of fertile tillers (12.80%), and 1000‐grain weight (12.87%) were all adversely affected. The comparatively smaller reduction in 1000‐grain weight relative to the decrease in filled grains suggests that the plants prioritized maintaining individual grain quality under stress, with yield loss primarily driven by a reduction in grain number. While certain genotypes, such as Kawther (G29), exhibited high susceptibility with approximately 54% yield loss, others like M1 (G32) and Shiroudi (G74) demonstrated relative tolerance, sustaining lower yield reductions (approximately 37% and 39%, respectively) (Table S4). This indicates that the reproductive stage, particularly grain filling, is highly sensitive to water deficit in rice. Interestingly, genotypes with lower yield reduction, such as M1 and Shiroudi, also showed smaller decreases in fertile tillers and panicle length, suggesting that maintenance of these traits under stress may contribute to their higher tolerance. These results align with the observations of Bhandari et al. (2023), who noted that drought tolerance in rice is often associated with the ability to sustain reproductive development and minimize spikelet sterility under water‐limited conditions.

**FIGURE 1:**
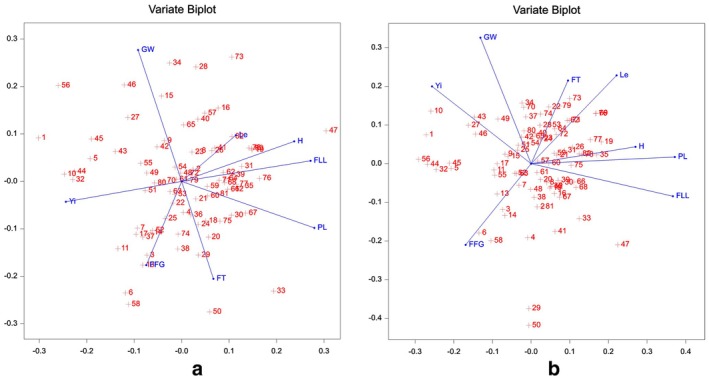
Variate Biplot visualizing relationships between agronomic traits and grain yield under normal irrigation (a) and terminal drought stress (b) conditions. Under both conditions, grain yield (Yi) clusters closely with filled grains per panicle (FFG) and 1000‐grain weight (GW), indicating their pivotal role in yield determination. In contrast, plant height (H), panicle length (PL), and flag leaf length (FLL) are positioned opposite to yield, suggesting a potential trade‐off under drought. High‐performing genotypes such as M1 (32), Sela‐Zodras (44), and Shiroudi (74) are located in the high‐yield sector of the biplot. Y: year; En: environment (normal vs. stress); G: genotype; R: replicate; df: degrees of freedom; CV: coefficient of variation; ns, * and **, no significance, significance at *p* ≤ 0.05, and *p* ≤ 0.01, respectively. (a) Normal condition (b) Stress condition. 1: A16; 2: Ak‐ypyk; 3: Amber albalaka; 4: Anbar33; 5: Atai‐1; 6: Barnamaj4; 7: BT7; 8: C10; 9: Cakmak; 10: D3; 11: Dijla; 12: Dollar; 13: Furat1; 14: Ghadeer; 15: Halibey; 16: Hikkan Hashimi; 17: HT1; 18: Iba; 19: IR28; 20: IR30; 21: IR36; 22: IR50; 23: IR58; 24: IR60; 25: IR64; 26: Iskander; 27: Jalal Abad; 28: Kapa; 29: Kawther; 30: Labypma; 31: LT2; 32: M1; 33: Manyas Yildizi; 34: Marjan; 35: Mis‐2013; 36: Mishkab1; 37: Mishkab2; 38: Mustakillik; 39: Okean; 40: Osmancik‐97; 41: Avangard; 42: Pasali; 43: QazNIIR‐7; 44: Sela‐Zodras; 45: Shalawangi 1; 46: Shalawangi 2; 47: Siyavar Hasimi; 48: Sumer; 49: Syl Sulu; 50: T85; 51: Tantana; 52: Tarona; 53: TosyaGunesi; 54: V20‐48(awn); 55: V20‐53‐2‐2; 56: V20‐8‐2; 57: Xazaz Hazar; 58: Yasmine; 59: Ahlami Tarom; 60: Abjiboji; 61: Bojar; 62: Binam; 63: Champa Boodar; 64: Hasansaraei; 65: Hasani; 66: Khazar; 67: Domzard; 68: Domsefid; 69: Domsiah; 70: Sahel; 71: Salari; 72: SangTarom; 73: Shahpasand; 74: Shiroudi; 75: Saleh; 76: Alikazemi; 77: Anbarboo; 78: Gharib; 79: Kadous; 80: Gilaneh; 81: Mohammadi; and 82: Hashemi. FFG, full‐filled grain per panicle; FLL, flag leaf length; FT, fertile tillers; GW, 1000‐Grain weight; H, height; Le, Kernel length; Yi, yield.

The correlation patterns were further elucidated using Biplot analysis, which visually reinforced the relationships among traits and genotypes across environments (Figure 1). Under both normal irrigation and terminal drought stress, grain yield consistently aligned closely with filled grains per panicle and 1000‐grain weight in the biplot space, confirming their pivotal role in yield stability under drought. In contrast, traits such as plant height, panicle length, and flag leaf length were positioned oppositely to yield, highlighting a potential adaptive trade‐off under water‐limited conditions. The biplot also clearly grouped high‐performing genotypes—notably M1, Sela‐Zodras, and Shiroudi—within the high‐yield and high‐stress‐tolerance sector, while sensitive genotypes clustered separately. This integrated graphical approach substantiates that selection for grain‐filling capacity and grain weight retention, coupled with a compact plant architecture, provides a robust phenotypic framework for identifying drought‐resilient rice lines.

**FIGURE 2:**
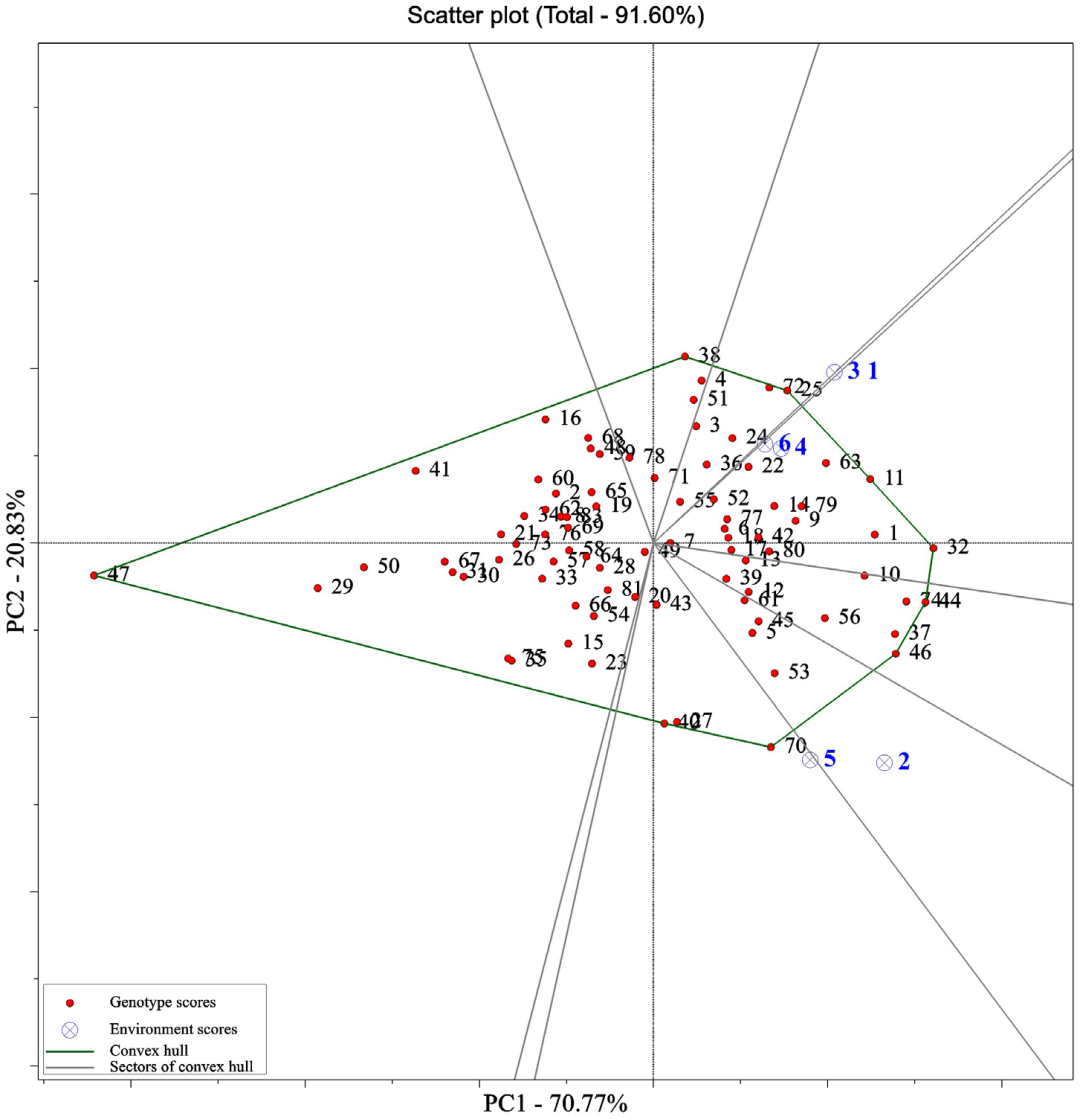
GGE‐biplot polygon view displaying genotype performance and mega‐environment classification across six environments. Genotypes at the vertices of the polygon (e.g., M1/G32, Sela‐Zodras/G44, Shalawangi 2/G46, Siyavar Hasimi/G47) are the best or poorest performers in one or more environments. Lines drawn from the biplot origin perpendicular to the polygon sides divide the biplot into sectors representing different mega‐environments. M1 (G32) is located farthest in the high‐yield direction, while Siyavar Hasimi (G47) is positioned in the low‐yield sector. Environmental scores include 1: Normal condition in year 2020; 2: Normal condition in year 2021; 3: Normal condition in year 2022; 4: Stress condition in year 2020; 5: Stress condition in year 2021; and 6: Stress condition in year 2022. Genotype scores include 1: A16; 2: Ak‐ypyk; 3: Amber albalaka; 4: Anbar33; 5: Atai‐1; 6: Barnamaj4; 7: BT7; 8: C10; 9: Cakmak; 10: D3; 11: Dijla; 12: Dollar; 13: Furat1; 14: Ghadeer; 15: Halibey; 16: Hikkan Hashimi; 17: HT1; 18: Iba; 19: IR28; 20: IR30; 21: IR36; 22: IR50; 23: IR58; 24: IR60; 25: IR64; 26: Iskander; 27: Jalal Abad; 28: Kapa; 29: Kawther; 30: Labypma; 31: LT2; 32: M1; 33: Manyas Yildizi; 34: Marjan; 35: Mis‐2013; 36: Mishkab1; 37: Mishkab2; 38: Mustakillik; 39: Okean; 40: Osmancik‐97; 41: Avangard; 42: Pasali; 43: QazNIIR‐7; 44: Sela‐Zodras; 45: Shalawangi 1; 46: Shalawangi 2; 47: Siyavar Hasimi; 48: Sumer; 49: Syl Sulu; 50: T85; 51: Tantana; 52: Tarona; 53: TosyaGunesi; 54: V20‐48(awn); 55: V20‐53‐2‐2; 56: V20‐8‐2; 57: Xazaz Hazar; 58: Yasmine; 59: Ahlami Tarom; 60: Abjiboji; 61: Bojar; 62: Binam; 63: Champa Boodar; 64: Hasansaraei; 65: Hasani; 66: Khazar; 67: Domzard; 68: Domsefid; 69: Domsiah; 70: Sahel; 71: Salari; 72: SangTarom; 73: Shahpasand; 74: Shiroudi; 75: Saleh; 76: Alikazemi; 77: Anbarboo; 78: Gharib; 79: Kadous; 80: Gilaneh; 81: Mohammadi and 82: Hashemi.

To decipher the patterns of yield variation and the principal effects of environment, an analysis was conducted using the GGE biplot approach. The GGE biplot analysis revealed that the first two principal components (PC1 and PC2) of the GGE model, which together represent the genotype main effect (G) plus genotype‐by‐environment interaction (GE), accounted for 70.77% and 20.83% of the grain yield variation, respectively, cumulatively explaining 91.60% of the total variability (Figure 2). Therefore, these two components were deemed suitable for interpreting the grain yield of the genotypes. A GGE biplot polygon was constructed to identify mega‐environments and superior genotypes. In this visualization, genotypes positioned farthest from the biplot origin are connected by straight lines, forming a polygon. Perpendicular lines are then drawn from the origin to the sides of this polygon, delineating the mega‐environments. Genotypes located at the polygon vertices represent the top‐performing cultivars within their respective environments (Yan et al. 2000).

Figure 2 displays the biplot of grain yield for 82 genotypes evaluated across six environments. Genotypes Siyavar Hasimi (G47), Sahel (G70), Shalawangi 2 (G46), Sela‐Zodras (G44), M1 (G32), Dijla (G11), IR64 (G25), and Mustakillik (G38), which constitute the polygon vertices and are situated at the greatest distance from the biplot center, were identified as either the best or the poorest performers in specific or all environments. Notably, Genotype M1 (G32) demonstrated the highest grain yield, emerging as the superior genotype, whereas Genotype Siyavar Hasimi (G47), positioned at the farthest distance from the center, was recognized as the lowest‐yielding genotype.

**FIGURE 3:**
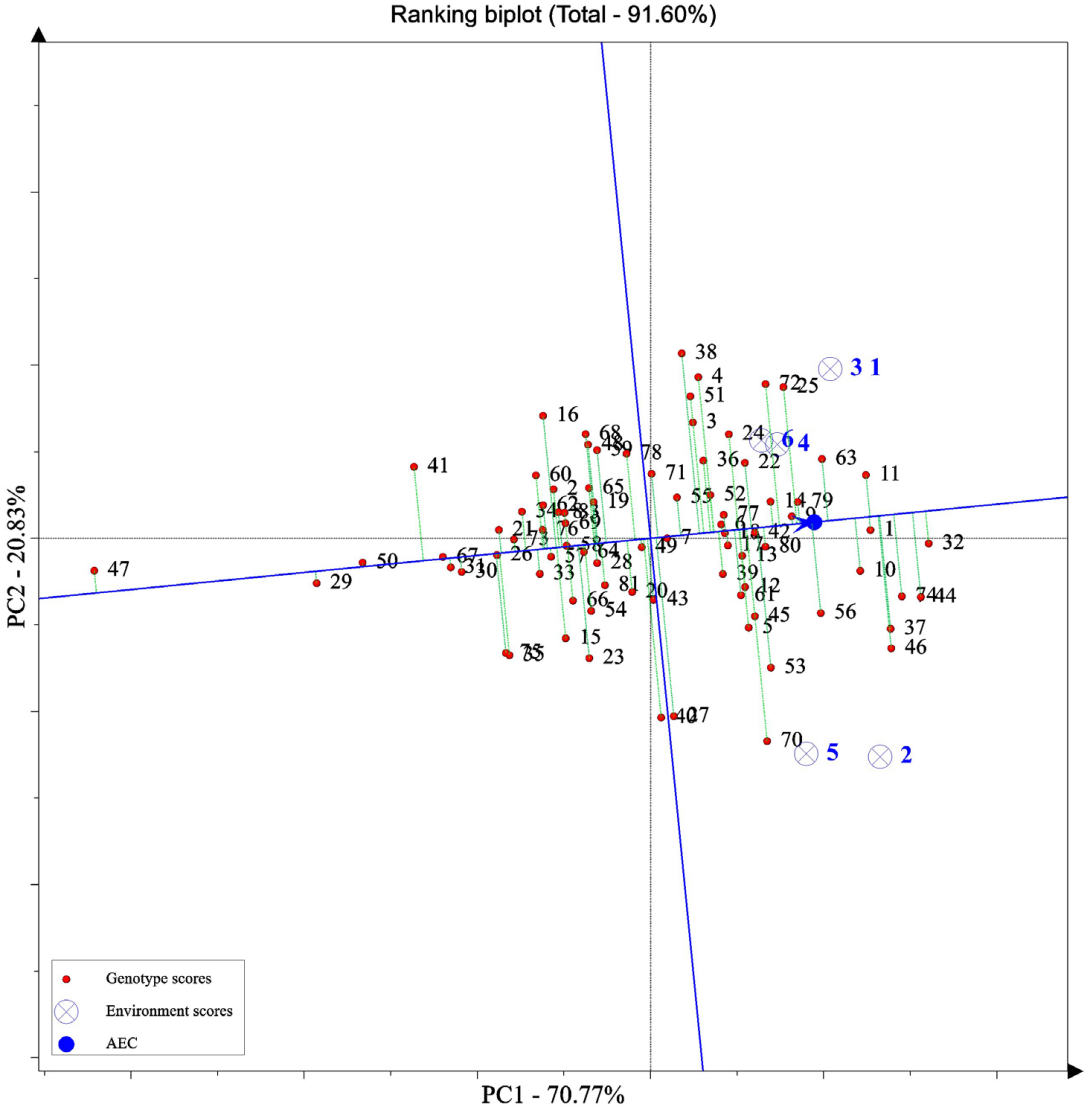
Two‐dimensional graph of average tester coordination (ATC) of the GGE‐biplot for simultaneous evaluation of stability and yield of genotypes. The biplot is based on the GGE model, with the ATC abscissa (horizontal line) representing the average environment. The vertical blue line indicates the mean yield across environments. Genotypes closer to the ATC line exhibit higher stability; those farther to the right along the ATC axis show higher yield. M1 (G32) is positioned near the ATC line and to the far right, indicating both high yield and stability. Environmental scores include 1: Normal condition in year 2020; 2: Normal condition in year 2021; 3: Normal condition in year 2022; 4: Stress condition in year 2020; 5: Stress condition in year 2021; and 6: Stress condition in year 2022. Genotype scores include 1: A16; 2: Ak‐ypyk; 3: Amber albalaka; 4: Anbar33; 5: Atai‐1; 6: Barnamaj4; 7: BT7; 8: C10; 9: Cakmak; 10: D3; 11: Dijla; 12: Dollar; 13: Furat1; 14: Ghadeer; 15: Halibey; 16: Hikkan Hashimi; 17: HT1; 18: Iba; 19: IR28; 20: IR30; 21: IR36; 22: IR50; 23: IR58; 24: IR60; 25: IR64; 26: Iskander; 27: Jalal Abad; 28: Kapa; 29: Kawther; 30: Labypma; 31: LT2; 32: M1; 33: Manyas Yildizi; 34: Marjan; 35: Mis‐2013; 36: Mishkab1; 37: Mishkab2; 38: Mustakillik; 39: Okean; 40: Osmancik‐97; 41: Avangard; 42: Pasali; 43: QazNIIR‐7; 44: Sela‐Zodras; 45: Shalawangi 1; 46: Shalawangi 2; 47: Siyavar Hasimi; 48: Sumer; 49: Syl Sulu; 50: T85; 51: Tantana; 52: Tarona; 53: TosyaGunesi; 54: V20‐48(awn); 55: V20‐53‐2‐2; 56: V20‐8‐2; 57: Xazaz Hazar; 58: Yasmine; 59: Ahlami Tarom; 60: Abjiboji; 61: Bojar; 62: Binam; 63: Champa Boodar; 64: Hasansaraei; 65: Hasani; 66: Khazar; 67: Domzard; 68: Domsefid; 69: Domsiah; 70: Sahel; 71: Salari; 72: SangTarom; 73: Shahpasand; 74: Shiroudi; 75: Saleh; 76: Alikazemi; 77: Anbarboo; 78: Gharib; 79: Kadous; 80: Gilaneh; 81: Mohammadi; and 82: Hashemi.

The Average Tester Coordinate (ATC) biplot was employed for the simultaneous assessment of genotype stability and yield. Based on this analysis, Genotype M1 (G32) exhibited high stability, as reflected by its proximity to the ATC abscissa (ideal stability line) while maintaining high yield, as indicated by its distance from the vertical blue line (Figure 3).

**FIGURE 4:**
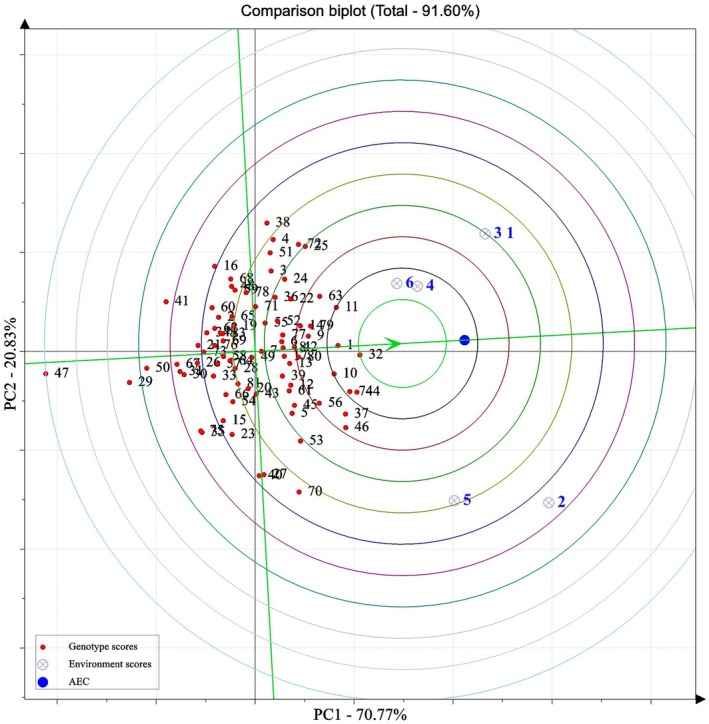
Biplot diagram for comparing the examined genotypes with the ideal genotype. Concentric circles are centered on the “ideal genotype,” which is defined as having the highest yield and perfect stability. Genotypes closer to the center (e.g., M1/G32) are considered elite, combining high performance with broad adaptability. Genotypes farther from the center (e.g., Siyavar Hasimi/G47) are less desirable. Environmental scores include 1: Normal condition in year 2020; 2: Normal condition in year 2021; 3: Normal condition in year 2022; 4: Stress condition in year 2020; 5: Stress condition in year 2021; and 6: Stress condition in year 2022. Genotype scores include 1: A16; 2: Ak‐ypyk; 3: Amber albalaka; 4: Anbar33; 5: Atai‐1; 6: Barnamaj4; 7: BT7; 8: C10; 9: Cakmak; 10: D3; 11: Dijla; 12: Dollar; 13: Furat1; 14: Ghadeer; 15: Halibey; 16: Hikkan Hashimi; 17: HT1; 18: Iba; 19: IR28; 20: IR30; 21: IR36; 22: IR50; 23: IR58; 24: IR60; 25: IR64; 26: Iskander; 27: Jalal Abad; 28: Kapa; 29: Kawther; 30: Labypma; 31: LT2; 32: M1; 33: Manyas Yildizi; 34: Marjan; 35: Mis‐2013; 36: Mishkab1; 37: Mishkab2; 38: Mustakillik; 39: Okean; 40: Osmancik‐97; 41: Avangard; 42: Pasali; 43: QazNIIR‐7; 44: Sela‐Zodras; 45: Shalawangi 1; 46: Shalawangi 2; 47: Siyavar Hasimi; 48: Sumer; 49: Syl Sulu; 50: T85; 51: Tantana; 52: Tarona; 53: TosyaGunesi; 54: V20‐48(awn); 55: V20‐53‐2‐2; 56: V20‐8‐2; 57: Xazaz Hazar; 58: Yasmine; 59: Ahlami Tarom; 60: Abjiboji; 61: Bojar; 62: Binam; 63: Champa Boodar; 64: Hasansaraei; 65: Hasani; 66: Khazar; 67: Domzard; 68: Domsefid; 69: Domsiah; 70: Sahel; 71: Salari; 72: SangTarom; 73: Shahpasand; 74: Shiroudi; 75: Saleh; 76: Alikazemi; 77: Anbarboo; 78: Gharib; 79: Kadous; 80: Gilaneh; 81: Mohammadi; and 82: Hashemi.

To facilitate a graphical assessment of the distance between the studied genotypes and ideal benchmark, concentric circles were superimposed onto the biplot, with the ideal genotype at their center (Figure 4). Genotypes located at or nearest to this central point are regarded as elite, possessing a desirable combination of high yield and stability. Genotype M1 (G32) was identified as the top genotype due to its shortest distance from the ideal genotype. Conversely, Genotype Siyavar Hasimi (G47), being the most distant from this benchmark, was concluded to be the least favorable genotype in this investigation.

To assess the genotypes' response to drought stress, yield‐based indices were employed. Evaluation of drought tolerance indices (Table S5) showed that grain yield under non‐stress conditions (Yn) ranged from 1020.9 kg/ha (Siyavar Hasimi) to 5937.8 kg/ha (M1), while under drought stress (Ys), it varied from 847.3 kg/ha (Siyavar Hasimi) to 3723.3 kg/ha (M1) (Table S5). This considerable disparity, observed both among genotypes and between the two environments, confirms a strong genetic potential for selection.

The Stress Susceptibility Index (SSI), which measures the relative yield reduction, ranged from 0.98 (T85) to 1.92 (IR58) (Table S5). Genotypes such as T85, with SSI values close to or below 1, are classified as tolerant, as their yield reduction was less than the average reduction across the population. Conversely, genotypes like IR58 and Dollar, with SSI values exceeding 1.8, are categorized as highly sensitive. However, interpreting SSI in isolation can be misleading. For instance, a genotype with inherently very low yield potential may exhibit a low SSI without being agronomically desirable. Therefore, integrating this index with yield performance data is essential for a meaningful assessment. The Tolerance Index (TOL) provided complementary information, with IR58 showing the lowest value (902.22) despite its high SSI, highlighting the complex relationship between relative and absolute measures of drought response (Table S5). The Stress Tolerance Index (STI) is widely recognized as a reliable criterion for identifying genotypes capable of high yield under both stress and non‐stress conditions (Fernandez 1992). In this study, genotypes M1 (No. 32, STI = 145.18), Sela‐Zodras (No. 44, STI = 143.27), and Shiroudi (No. 74, STI = 140.17) displayed the highest STI values. These genotypes combined high yield under optimal conditions (5937.8, 5846.7, and 5799.4 kg/ha, respectively) with commendable yield under drought stress (3723.3, 3687.1, and 3528.2 kg/ha, respectively) (Table S5). This consistent yield establishes them as ideal candidates, characterized by high yield stability and significant drought tolerance. The Geometric Mean Productivity (GMP) and Mean Productivity (MP) indices corroborated these findings, ranking the aforementioned genotypes at the top. The Harmonic Mean (HM) and Abiotic Tolerance Index (ATI) provided robust validation of genotype yield. The strong correlation between these indices and STI (M1: HM = 4576.77, ATI = 6291387.79; Shiroudi: HM = 4387.32, ATI = 6207754.41) confirms the consistency of selection across different mathematical approaches. The Relative Drought Index (RDI) further supported these findings, with high‐yielding genotypes showing values above 1.0, indicating better‐than‐average yield under stress. The high positive correlation typically observed between STI, GMP, MP, and yield in both environments (data not shown) confirms that these indices are powerful tools for simultaneous selection for both yield potential and stability.

Based on the various indices, the genotypes can be categorized into distinct groups:

– Tolerant and high‐yielding genotypes:

This group includes genotypes M1, Sela‐Zodras, Shiroudi, Mishkab2, and D3, which exhibit high STI and GMP with moderate SSI values. These genotypes represent the final candidates for potential release as drought‐tolerant cultivars.

– Sensitive yet high‐yielding under non‐stress conditions: Genotypes such as Kadous (No. 79) possess very high yield under optimal conditions (5599 kg/ha) but suffer a severe yield penalty under stress (YSI = 2.07). These are suitable for cultivation in regions without water limitations.
– Tolerant genotypes with moderate yield: Genotypes like Okean (No. 39) and QazNIIR‐7 (No. 43), while not exhibiting the highest overall yield, show respectable yield under stress and can be valued as genetic resources for tolerance in breeding programs.
– Sensitive and low‐yielding genotypes: Genotypes T85 (No. 50), Kawther (No. 29), and Siyavar Hasimi (No. 47), which recorded the lowest values for yield and stability indices, are identified as sensitive.

The results of this study demonstrated that genotypes such as M1 (G32), Sela‐Zodras (G44), and Shiroudi (G74) exhibited superior yield under both normal and drought stress conditions, as reflected by their high STI, GMP, and MP values. These genotypes not only maintained relatively high yield under stress but also showed moderate stress susceptibility indices (SSI), indicating a balanced response to water limitation. The consistency of these genotypes across multiple indices suggests that they possess complementary physiological and genetic mechanisms for drought adaptation, such as improved water‐use efficiency, osmotic adjustment, or deeper root systems. Similar findings have been reported by Sabouri et al. (2022) and Kandel et al. (2022), who emphasized that genotypes with high STI and GMP are ideal candidates for cultivation in drought‐prone environments due to their stability and resilience.

The correlation matrix (Table 4) further indicated a high degree of multicollinearity (*r* > 0.98) among several key indices, including Mean Productivity (MP), Geometric Mean Productivity (GMP), Stress Tolerance Index (STI), and Yield Index (YI). This suggests a significant redundancy, implying that these indices convey overlapping information regarding genotypic performance (Table 4).

**TABLE 4:** Correlation matrix among drought tolerance and susceptibility indices for the 82 rice genotypes evaluated under normal and terminal drought stress conditions.

	Yn	Ys	SSI	TOL	MP	GMP	STI	YI	YSI	HM	RDI	ATI
Yn	1.00											
Ys	0.90	1.00										
SSI	0.05	0.47	1.00									
TOL	0.78	0.43	−0.56	1.00								
MP	0.98	0.96	0.23	0.65	1.00							
GMP	0.97	0.98	0.28	0.61	0.99	1.00						
STI	0.98	0.96	0.23	0.65	0.99	1.00	1.00					
YI	0.90	0.99	0.47	0.42	0.96	0.98	0.96	1.00				
YSI	0.04	0.47	1.00	−0.57	0.22	0.28	0.22	0.47	1.00			
HM	0.95	0.99	0.34	0.56	0.99	0.99	0.99	0.99	0.33	1.00		
RDI	0.05	0.47	0.99	−0.56	0.23	0.29	0.23	0.48	1.00	0.34	1.00	
ATI	0.94	0.73	−0.19	0.91	0.88	0.85	0.88	0.72	−0.20	0.82	−0.19	1.00

*Note:* Grain yield under non‐stress (Yn) and stress (Ys) conditions, Stress Susceptibility Index (SSI), Tolerance Index (TOL), Mean Productivity (MP), Geometric Mean Productivity (GMP), Stress Tolerance Index (STI), Yield Index (YI), Yield Stability Index (YSI), Harmonic Mean (HM), Relative Drought Index (RDI), and Abiotic Tolerance Index (ATI).

The principal component analysis (PCA) effectively reduced the dimensionality of the dataset. The scree plot and eigenvalues revealed that only the first two principal components (PCs) possessed eigenvalues greater than 1, collectively accounting for 99.23% of the total variance within the data. The first principal component (PC1) alone explained 68.20% of the variance, while the second component (PC2) contributed an additional 31.04%. This exceptionally high cumulative variance explained by the first two components underscores the presence of a strong, underlying structure in the data and demonstrates that the complex interactions of the 12 indices can be effectively captured and interpreted within a simplified, two‐dimensional latent factor space (Figure S1).

**FIGURE 5:**
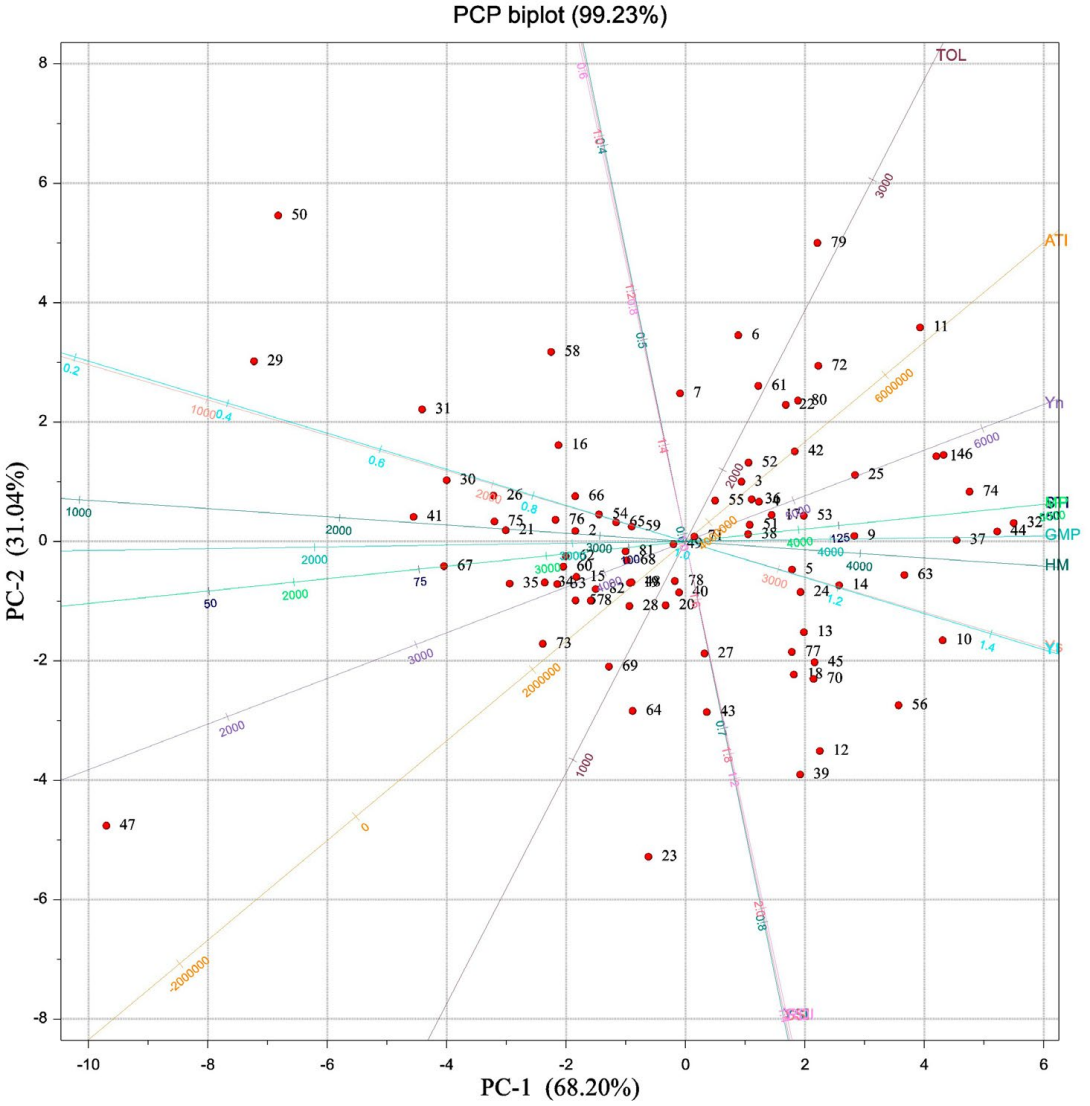
Biplot principal component analysis (PCA) of drought tolerance indices. The biplot illustrates the relationships among 12 drought indices across 82 rice genotypes. PC1 (68.20% of variance) represents general yield performance, with high loadings from MP, GMP, STI, and HM. PC2 (31.04% of variance) distinguishes stress susceptibility (positive loadings: SSI, YSI, RDI) from stress tolerance (negative loadings: TOL, ATI). Genotypes positioned in the upper‐left quadrant exhibit high stress tolerance, while those in the lower‐right show high yield potential under non‐stress conditions. A16; 2: Ak‐ypyk; 3: Amber albalaka; 4: Anbar33; 5: Atai‐1; 6: Barnamaj4; 7: BT7; 8: C10; 9: Cakmak; 10: D3; 11: Dijla; 12: Dollar; 13: Furat1; 14: Ghadeer; 15: Halibey; 16: Hikkan Hashimi; 17: HT1; 18: Iba; 19: IR28; 20: IR30; 21: IR36; 22: IR50; 23: IR58; 24: IR60; 25: IR64; 26: Iskander; 27: Jalal Abad; 28: Kapa; 29: Kawther; 30: Labypma; 31: LT2; 32: M1; 33: Manyas Yildizi; 34: Marjan; 35: Mis‐2013; 36: Mishkab1; 37: Mishkab2; 38: Mustakillik; 39: Okean; 40: osmancik‐97; 41: Avangard; 42: Pasali; 43: QazNIIR‐7; 44: Sela‐Zodras; 45: Shalawangi 1; 46: Shalawangi 2; 47: Siyavar Hasimi; 48: Sumer; 49: Syl Sulu; 50: T85; 51: Tantana; 52: Tarona; 53: TosyaGunesi; 54: V20‐48(awn); 55: V20‐53‐2‐2; 56: V20‐8‐2; 57: Xazaz Hazar; 58: Yasmine; 59: Ahlami Tarom; 60: Abjiboji; 61: Bojar; 62: Binam; 63: Champa Boodar; 64: Hasansaraei; 65: Hasani; 66: Khazar; 67: Domzard; 68: Domsefid; 69: Domsiah; 70: Sahel; 71: Salari; 72: SangTarom; 73: Shahpasand; 74: Shiroudi; 75: Saleh; 76: Alikazemi; 77: Anbarboo; 78: Gharib; 79: Kadous; 80: Gilaneh; 81: Mohammadi and 82: Hashemi. Grain yield under non stress (Yn) and stress (Ys) conditions, Stress Susceptibility Index (SSI), Tolerance Index (TOL), Mean Productivity (MP), Geometric Mean Productivity (GMP), Stress Tolerance Index (STI), Yield Index (YI), Yield Stability Index (YSI), Harmonic Mean (HM), Relative Drought Index (RDI), and Abiotic Tolerance Index (ATI).

PC1 was characterized by positive and relatively uniform loadings across all measured variables. The highest loadings were associated with GMP (0.349), MP (0.348), STI (0.348), and Harmonic Mean (HM) (0.348). So, PC1 can be interpreted as a “General Performance and Yield Potential” axis. Genotypes with high scores on PC1 are likely to exhibit superior overall yield across both non‐stress and drought‐stress environments. In contrast, PC2 displayed a clear dichotomy in variable loadings. The Stress Susceptibility Index (SSI), Yield Stability Index (YSI), and Rainfall Drought Index (RDI) loaded positively and strongly (approximately 0.49–0.49), whereas the Tolerance Index (TOL) and Abiotic Tolerance Index (ATI) exhibited significant negative loadings (−0.41 and −0.25, respectively). PC2 showed strong positive loadings for SSI, YSI, and RDI, indicating that high values along this axis reflect greater yield reduction under stress (i.e., susceptibility). Therefore, genotypes with low PC2 scores exhibit better stress tolerance and yield stability. Genotypes with high PC2 scores demonstrate greater resilience and maintained yield under drought conditions, effectively distinguishing them from those merely possessing high yield potential (Figure 5).

The high correlation among MP, GMP, STI, and YI (*r* > 0.98) indicates that these indices provide overlapping information and can be used interchangeably for selecting high‐yielding genotypes under both stress and non‐stress conditions. However, the distinct loading pattern of SSI and YSI on PC2 in the PCA highlights their utility in distinguishing stress‐responsive genotypes from those with high yield potential alone. The use of multivariate methods such as GGE biplot and PCA allowed for a more integrated assessment of genotype performance and stability, facilitating the identification of ideal genotypes like M1, which combined high yield with broad adaptability. Similar approaches have been advocated by Yan and Kang (2002) and Nagaraju et al. (2023) for effective selection in multi‐environment trials.

The identification of drought‐tolerant genotypes like M1, Sela‐Zodras, and Shiroudi provides valuable genetic resources for breeding programs aimed at enhancing climate resilience in rice. These genotypes can be directly used as donors in hybridization programs or deployed in regions experiencing terminal drought stress. Furthermore, the strong association between STI and yield under stress suggests that this index can be effectively used for early‐generation screening. Future studies should focus on elucidating the molecular and physiological mechanisms underlying the drought tolerance of these genotypes, as well as validating their yield in diverse agro‐ecological zones to ensure broad adaptability.

The panel used in this study included genotypes from diverse geographical regions (Central and Western Asia, IRRI) and represented a mix of landraces, improved varieties, and breeding lines. While a detailed analysis of population structure was beyond the scope of this study, we observed that drought‐tolerant genotypes were not confined to a single origin or type. For instance, M1 (Iraq) and Sela‐Zodras (Afghanistan) are both landraces from arid regions, suggesting that local adaptation has conferred inherent drought tolerance. In contrast, some modern varieties from IRRI showed high sensitivity, possibly due to selection under optimal conditions. This underscores the importance of targeted germplasm mining—prioritizing materials from stress‐prone regions—as a strategy for enhancing drought resilience. Future studies incorporating genomic data could elucidate the specific alleles and haplotypes associated with the tolerance observed in these accessions.

Conducting yield trials under drought stress across multiple environments is resource‐intensive. Our correlation and PCA results suggest that certain morpho‐physiological traits could serve as effective proxies for drought tolerance screening. Specifically, the number of filled grains per panicle and fertile tiller count showed consistent positive correlations with yield under both conditions, while plant height and panicle length were negatively associated with yield under stress. This indicates that compact, high‐tillering genotypes with efficient grain filling are better adapted to terminal drought. Traits such as 1000‐grain weight also maintained a strong positive correlation with yield under stress, suggesting that mechanisms preserving grain size contribute to drought resilience. Incorporating these easily measurable traits into early‐generation screening could reduce the cost and time of breeding programs while maintaining selection accuracy, a strategy supported by recent studies (Bhandari et al. 2023; Abbas et al. 2025).

## 4 Conclusion

This study successfully identified drought‐tolerant and high‐yielding rice genotypes using a comprehensive approach integrating quantitative yield‐based indices and multivariate analyses. Terminal drought stress was confirmed to be a major constraint, causing a substantial 39.58% reduction in grain yield, primarily driven by a decrease in filled grains per panicle.

The combined analysis of variance revealed significant genetic diversity among the 82 genotypes evaluated, along with notable genotype‐by‐environment interactions, underscoring the necessity for multi‐environment testing to identify stable performers. Among the suite of drought indices evaluated, the Stress Tolerance Index (STI), Geometric Mean Productivity (GMP), and Mean Productivity (MP) proved to be the most effective and reliable criteria for simultaneous selection for high yield potential and drought tolerance. This was confirmed by their high collinearity (*r* > 0.98) and central role in the Principal Component Analysis.

The multivariate GGE biplot analysis consistently highlighted genotypes M1 (G32), Sela‐Zodras (G44), and Shiroudi (G74) as superior and ideal genotypes. These genotypes not only exhibited high yields under both normal and terminal drought stress conditions but also demonstrated remarkable yield stability. Specifically, M1 was identified as the most ideal genotype, combining the highest yield with superior stability across environments.

In conclusion, the elite genotypes M1, Sela‐Zodras, and Shiroudi represent invaluable genetic resources. They are highly recommended for direct cultivation in drought‐prone regions to stabilize rice production and, equally importantly, for use as donor parents in breeding programs aimed at enhancing climate resilience and developing the next generation of drought‐tolerant rice varieties. The study solidifies the recommendation of STI, GMP, and MP as key selection indices for efficient screening in such breeding endeavors.

## Funding

The authors have nothing to report.

## Conflicts of Interest

The authors declare no conflicts of interest.

## Supporting information


**Table S1:** Meteorological data (temperature and rainfall) during the experimental period (2020–2022) at the Rice Research Institute of Iran, Rasht.
**Table S2:** Soil moisture status during the experimental period (2020–2022) after withholding irrigation.
**Table S3:** Mean and variability of height, fertile tiller, panicle length and flag leaf length in the studied rice genotypes under drought stress.
**Table S4:** Mean and variability of full filled grain per panicle, yield, 1000‐grain weight and kernel length in the studied rice genotypes under drought stress.
**Table S5:** Tolerance and susceptibility index of 82 rice genotypes under conditions of drought stress.
**Figure S1:** Scree plot and variance explained of Principal Component Analysis (PCA).

## Data Availability

The data that supports the findings of this study are available in the Supporting Information of this article.
