# Supplementary material for: Quantitative Indices for Drought Tolerance in Rice: Leveraging Genetic Resources for Climate‐Resilient Breeding
Source: Plant Environ Interact. 2026 May 14;7(3):e70145. doi: 10.1002/pei3.70145 (PMC13173357; doi:10.1002/pei3.70145)
Supplement: Supplementary file 1 — Table S1: Meteorological data (temperature and rainfall) during the experimental period (2020–2022) at the Rice Research Institute of Iran, Rasht. Table S2: Soil moisture status during the experimental period (2020–2022) after withholding irrigation. Table S3: Mean and variability of height, fertile tiller, panicle length and flag leaf length in the studied rice genotypes under drought stress. Table S4: Mean and variability of full filled grain per panicle, yield, 1000‐grain weight and kernel length in the studied rice genotypes under drought stress. Table S5: Tolerance and susceptibility index of 82 rice genotypes under conditions of drought stress. Figure S1: Scree plot and variance explained of Principal Component Analysis (PCA). [file PEI3-7-e70145-s001.docx]

| Table S1. Meteorological data (temperature and rainfall) during the experimental period (2020–2022) at the Rice Research Institute of Iran, Rasht. | | | | | | |
| --- | --- | --- | --- | --- | --- | --- |
| Month | 2020 | | 2021 | | 2022 | |
|  | Precipitation  Mean  (mm) | Temperature  Mean  (Celsius) | Precipitation  Mean  (mm) | Temperature  Mean  (Celsius) | Precipitation  Mean  (mm) | Temperature  Mean  (Celsius) |
| April | 167.2 | 13.4 | 15.2 | 17 | 14.2 | 17.9 |
| May | 23.3 | 20.9 | 46.8 | 18 | 41.9 | 21 |
| June | 0.4 | 25.3 | 1.2 | 25.2 | 5.3 | 26 |
| July | 26.6 | 27 | 37 | 25.6 | 104.1 | 26.5 |
| August | 158.1 | 23.7 | 15.9 | 26.8 | 7 | 28.1 |
| September | 32.9 | 24 | 129.5 | 24.1 | 78.1 | 23.4 |

| Table S2. Soil moisture status during the experimental period (2020–2022) after withholding irrigation. | | | | | | |
| --- | --- | --- | --- | --- | --- | --- |
|  | 2020 | | 2021 | | 2022 | |
|  | Weighted Soil Moisture (%) | Soil water potential (kPa) | Weighted Soil Moisture (%) | Soil water potential (kPa) | Weighted Soil Moisture (%) | Soil water potential (kPa) |
| 10 Days after withholding irrigation | 55 | -0.8 | 56 | -0.7 | 58 | -0.6 |
| 15 Days after withholding irrigation | 48 | -28 | 49 | -20 | 47 | -19 |
| 20 Days after withholding irrigation | 38 | -168 | 38 | -164 | 38 | -158 |
| 25 Days after withholding irrigation | 32 | -501 | 32 | -418 | 33 | -521 |
| 30 Days after withholding irrigation | 25 | -1312 | 22 | -1356 | 26 | -1370 |

| Table S3. mean and variability of height, fertile tiller, panicle length and flag leaf length in the studied rice genotypes under drought stress | | | | | | | | | | | | |
| --- | --- | --- | --- | --- | --- | --- | --- | --- | --- | --- | --- | --- |
| **G** | **Height (cm)** | | | **Fertile tiller** | | | **Panicle Length (cm)** | | | **Flag Leaf Length (cm)** | | |
|  | **Normal** | **Stress** | **Variation  (%)** | **Normal** | **Stress** | **Variation  (%)** | **Normal** | **Stress** | **Variation  (%)** | **Normal** | **Stress** | **Variation  (%)** |
| **1** | 106.58 | 95.27 | -10.61 | 10.74 | 9.97 | -7.17 | 18 | 13.86 | -23 | 18.67 | 14.22 | -23.84 |
| **2** | 141.74 | 123.91 | -12.58 | 10.59 | 9.2 | -13.13 | 26.4 | 21.64 | -18.03 | 37.47 | 26.14 | -30.24 |
| **3** | 108.16 | 94.09 | -13.01 | 14.39 | 11.79 | -18.07 | 26.62 | 20.36 | -23.52 | 32.19 | 25.1 | -22.03 |
| **4** | 123.8 | 110.05 | -11.11 | 10.59 | 9.31 | -12.09 | 36.34 | 29.6 | -18.55 | 33.2 | 27.66 | -16.69 |
| **5** | 124.2 | 113.12 | -8.92 | 11.34 | 10.53 | -7.14 | 17.57 | 14.16 | -19.41 | 26.01 | 17.8 | -31.56 |
| **6** | 111.96 | 98.22 | -12.27 | 16.14 | 12.94 | -19.83 | 26.96 | 18.58 | -31.08 | 26.17 | 21.31 | -18.57 |
| **7** | 97.58 | 86.89 | -10.96 | 15.43 | 13.17 | -14.65 | 26.96 | 22.36 | -17.06 | 26.33 | 23.24 | -11.74 |
| **8** | 117.69 | 103.32 | -12.21 | 9.83 | 8.46 | -13.94 | 34.48 | 28.08 | -18.56 | 30.37 | 24.82 | -18.27 |
| **9** | 140.85 | 123.19 | -12.54 | 12.3 | 11.27 | -8.37 | 26.94 | 21.52 | -20.12 | 36.23 | 25.44 | -29.78 |
| **10** | 111.96 | 100.69 | -10.07 | 15.21 | 13.78 | -9.4 | 19.11 | 15.88 | -16.9 | 20.37 | 15.7 | -22.93 |
| **11** | 107.27 | 94.76 | -11.66 | 16.06 | 15.04 | -6.35 | 27.69 | 21.72 | -21.56 | 26.99 | 22.43 | -16.9 |
| **12** | 166.07 | 147.91 | -10.94 | 13.21 | 11.91 | -9.84 | 34.06 | 25.99 | -23.69 | 42.27 | 32.99 | -21.95 |
| **13** | 107.67 | 94 | -12.7 | 15.26 | 13.42 | -12.06 | 26.88 | 20.86 | -22.4 | 31.74 | 24.41 | -23.09 |
| **14** | 110.18 | 98.64 | -10.47 | 12.61 | 11.49 | -8.88 | 29.77 | 23.77 | -20.15 | 36.26 | 29.87 | -17.62 |
| **15** | 127.22 | 114.39 | -10.08 | 11.7 | 10.5 | -10.26 | 20.08 | 16.68 | -16.93 | 38.08 | 24.82 | -34.82 |
| **16** | 147.4 | 131.25 | -10.96 | 8.48 | 7.76 | -8.49 | 27.97 | 21.52 | -23.06 | 42.27 | 28.19 | -33.31 |
| **17** | 97.94 | 91 | -7.09 | 16.5 | 14.17 | -14.12 | 28.09 | 18.53 | -34.03 | 26.33 | 24.24 | -7.94 |
| **18** | 127 | 113.22 | -10.85 | 13.08 | 11.67 | -10.78 | 35.31 | 29.16 | -17.42 | 40.54 | 30.76 | -24.12 |
| **19** | 147.62 | 130.58 | -11.54 | 13.14 | 11.68 | -11.11 | 37.97 | 31.09 | -18.12 | 40.22 | 32.49 | -19.22 |
| **20** | 151.42 | 133.79 | -11.64 | 16.26 | 13.6 | -16.36 | 31.77 | 24.86 | -21.75 | 33.7 | 24.72 | -26.65 |
| **21** | 114.54 | 101.96 | -10.98 | 15.88 | 13.33 | -16.06 | 31.32 | 25.27 | -19.32 | 31.41 | 25.26 | -19.58 |
| **22** | 113.31 | 101.18 | -10.71 | 15.81 | 14.2 | -10.18 | 30.97 | 24.11 | -22.15 | 32.72 | 25.52 | -22 |
| **23** | 111.78 | 103.45 | -7.45 | 13.88 | 11.47 | -17.36 | 26.66 | 22 | -17.48 | 33.13 | 26.17 | -21.01 |
| **24** | 129.54 | 114.4 | -11.69 | 17.32 | 15.18 | -12.36 | 29.61 | 23.71 | -19.93 | 35.79 | 26.21 | -26.77 |
| **25** | 133.89 | 118.89 | -11.2 | 16.54 | 15.07 | -8.89 | 27.92 | 22.07 | -20.95 | 35.04 | 25.32 | -27.74 |
| **26** | 134.78 | 121.78 | -9.65 | 13.26 | 10.02 | -24.43 | 31.42 | 25.69 | -18.24 | 39.42 | 29.36 | -25.52 |
| **27** | 124.91 | 111.99 | -10.34 | 12.5 | 11.04 | -11.68 | 24.94 | 20.02 | -19.73 | 27.34 | 17.31 | -36.69 |
| **28** | 153 | 134.67 | -11.98 | 10.52 | 9.34 | -11.22 | 27.8 | 22.52 | -18.99 | 38.08 | 26.99 | -29.12 |
| **29** | 125.65 | 102.89 | -18.11 | 11.88 | 7.39 | -37.79 | 28.66 | 19.26 | -32.8 | 32 | 25.09 | -21.59 |
| **30** | 134.6 | 115.65 | -14.08 | 15.23 | 12.64 | -17.01 | 33.19 | 26.03 | -21.57 | 33.28 | 25.43 | -23.59 |
| **31** | 162 | 145.89 | -9.94 | 16.86 | 15.61 | -7.41 | 29.98 | 21.11 | -29.59 | 37.33 | 27.29 | -26.9 |
| **32** | 117.89 | 106.95 | -9.28 | 13.12 | 11.36 | -13.41 | 17.82 | 14.69 | -17.56 | 28.29 | 17.46 | -38.28 |
| **33** | 144 | 135 | -6.25 | 18.28 | 13.83 | -24.34 | 39.8 | 26.82 | -32.61 | 46 | 34.38 | -25.26 |
| **34** | 125.16 | 112.48 | -10.13 | 13.54 | 12.02 | -11.23 | 23.68 | 17.14 | -27.62 | 32.13 | 22.66 | -29.47 |
| **35** | 150.16 | 134.92 | -10.15 | 15.68 | 14.21 | -9.37 | 30.91 | 26.11 | -15.53 | 43.96 | 32.24 | -26.66 |
| **36** | 114 | 100.06 | -12.23 | 13.14 | 11.57 | -11.95 | 31.32 | 26.11 | -16.63 | 37.21 | 29.9 | -19.65 |
| **37** | 107.22 | 96.06 | -10.41 | 16.19 | 14.82 | -8.46 | 29.88 | 24.42 | -18.27 | 27.3 | 23.87 | -12.56 |
| **38** | 103.09 | 90.18 | -12.52 | 14.94 | 12.2 | -18.34 | 30.44 | 24.03 | -21.06 | 37.46 | 29.22 | -22 |
| **39** | 152.09 | 135.07 | -11.19 | 13.5 | 12.79 | -5.26 | 30.18 | 24.41 | -19.12 | 50.76 | 38.89 | -23.38 |
| **40** | 134.65 | 118.79 | -11.78 | 14.17 | 12.23 | -13.69 | 26.59 | 21.68 | -18.47 | 45.81 | 31.02 | -32.29 |
| **41** | 156.82 | 134.31 | -14.35 | 8.46 | 6.43 | -24 | 25.47 | 20.3 | -20.3 | 39.66 | 26.77 | -32.5 |
| **42** | 136.87 | 122.35 | -10.61 | 14.01 | 12.21 | -12.85 | 26.74 | 20.62 | -22.89 | 33.99 | 27.51 | -19.06 |
| **43** | 100.36 | 89.82 | -10.5 | 14.59 | 12.98 | -11.03 | 24.63 | 19.26 | -21.8 | 24.61 | 19.61 | -20.32 |
| **44** | 114.85 | 100.52 | -12.48 | 11.21 | 9.84 | -12.22 | 20.36 | 15.78 | -22.5 | 24.73 | 17.73 | -28.31 |
| **45** | 113.54 | 98.35 | -13.38 | 9.61 | 8.46 | -11.97 | 21.14 | 17.88 | -15.42 | 26.86 | 19.52 | -27.33 |
| **46** | 112.34 | 100.06 | -10.93 | 9.12 | 8.26 | -9.43 | 23.14 | 18.98 | -17.98 | 40.02 | 26 | -35.03 |
| **47** | 130.02 | 112.49 | -13.48 | 9.06 | 8.03 | -11.37 | 35.82 | 28.89 | -19.35 | 51.77 | 31.81 | -38.56 |
| **48** | 113.07 | 98.19 | -13.16 | 11.08 | 9.19 | -17.06 | 30.42 | 23.79 | -21.79 | 33.8 | 26.67 | -21.09 |
| **49** | 106.91 | 95.38 | -10.78 | 15.08 | 13.74 | -8.89 | 27.34 | 21.38 | -21.8 | 25.87 | 15.92 | -38.46 |
| **50** | 123.85 | 108.56 | -12.35 | 15.43 | 6.72 | -56.45 | 29.22 | 21.29 | -27.14 | 42.33 | 28.73 | -32.13 |
| **51** | 95.85 | 83.02 | -13.39 | 15.03 | 13.76 | -8.45 | 26.62 | 21.97 | -17.47 | 32.06 | 24.73 | -22.86 |
| **52** | 100.05 | 85.81 | -14.23 | 15.81 | 15.06 | -4.74 | 30.13 | 23.93 | -20.58 | 31.22 | 25.42 | -18.58 |
| **53** | 108.76 | 97.46 | -10.39 | 15.14 | 13.74 | -9.25 | 30.24 | 25.32 | -16.27 | 34.22 | 27.51 | -19.61 |
| **54** | 101.82 | 90.31 | -11.3 | 13.48 | 11.9 | -11.72 | 26.82 | 21.27 | -20.69 | 36.59 | 26.63 | -27.22 |
| **55** | 118.6 | 99.81 | -15.84 | 11.54 | 9.21 | -20.19 | 20.89 | 16.86 | -19.29 | 30.86 | 22.76 | -26.25 |
| **56** | 111.4 | 101.36 | -9.01 | 8.59 | 8.02 | -6.64 | 13.99 | 12.12 | -13.37 | 27.67 | 17.16 | -37.98 |
| **57** | 133 | 118 | -11.28 | 10.97 | 8.4 | -23.43 | 31.41 | 24.91 | -20.69 | 37.94 | 27.37 | -27.86 |
| **58** | 104.05 | 95.34 | -8.37 | 15.08 | 14.83 | -1.66 | 24.5 | 17.17 | -29.92 | 27.17 | 21.02 | -22.64 |
| **59** | 163.49 | 148.31 | -9.28 | 14.7 | 13.2 | -10.2 | 30.23 | 23.97 | -20.71 | 31.32 | 24.19 | -22.77 |
| **60** | 163.2 | 146.4 | -10.29 | 15.28 | 12.76 | -16.49 | 31.44 | 24.99 | -20.52 | 29.78 | 23.33 | -21.66 |
| **61** | 128.54 | 113.51 | -11.69 | 12.48 | 10.68 | -14.42 | 32.97 | 25.89 | -21.47 | 32.12 | 25.19 | -21.58 |
| **62** | 171 | 149.78 | -12.41 | 14.79 | 13.88 | -6.15 | 34.41 | 26.61 | -22.67 | 27.78 | 22.06 | -20.59 |
| **63** | 161.18 | 140.75 | -12.68 | 13.26 | 9.23 | -30.39 | 32.3 | 27.1 | -16.1 | 27.94 | 21.39 | -23.44 |
| **64** | 157.27 | 139.76 | -11.13 | 15.9 | 13.94 | -12.33 | 31.38 | 24.98 | -20.4 | 31.8 | 23.4 | -26.42 |
| **65** | 139.4 | 123.25 | -11.59 | 13.7 | 12.9 | -5.84 | 31.12 | 24.33 | -21.82 | 32.57 | 24.8 | -23.86 |
| **66** | 139.02 | 123.82 | -10.93 | 14.06 | 12.52 | -10.95 | 32.99 | 26 | -21.19 | 37.42 | 30.14 | -19.45 |
| **67** | 166.27 | 147.59 | -11.23 | 16.66 | 13.73 | -17.59 | 29.34 | 23.66 | -19.36 | 36.91 | 26.11 | -29.26 |
| **68** | 173.91 | 156.27 | -10.14 | 13.63 | 10.92 | -19.88 | 26.99 | 22.71 | -15.86 | 36.86 | 29.77 | -19.23 |
| **69** | 157.14 | 148.77 | -5.33 | 15.54 | 14.18 | -8.75 | 31.99 | 25.61 | -19.94 | 40.63 | 32.33 | -20.43 |
| **70** | 110 | 95.17 | -13.48 | 16.23 | 14.51 | -10.6 | 29.42 | 23.9 | -18.76 | 28.41 | 23.69 | -16.61 |
| **71** | 168.22 | 150.89 | -10.3 | 16.32 | 14.96 | -8.33 | 30.96 | 23.36 | -24.55 | 30.66 | 26.41 | -13.86 |
| **72** | 127.85 | 113.47 | -11.25 | 13.37 | 11.61 | -13.16 | 33.71 | 26.93 | -20.11 | 32.82 | 27.79 | -15.33 |
| **73** | 165.27 | 152.92 | -7.47 | 13.79 | 11.47 | -16.82 | 29.33 | 23.82 | -18.79 | 34.93 | 27.24 | -22.02 |
| **74** | 123.74 | 107.47 | -13.15 | 17.37 | 16.9 | -2.71 | 34.59 | 29.27 | -15.38 | 31.48 | 25.43 | -19.22 |
| **75** | 134.11 | 119.95 | -10.56 | 16.61 | 15.27 | -8.07 | 32.31 | 26.6 | -17.67 | 33.72 | 26.83 | -20.43 |
| **76** | 154.8 | 136.82 | -11.61 | 16.7 | 15.28 | -8.5 | 35.17 | 27.29 | -22.41 | 40.46 | 29.87 | -26.17 |
| **77** | 164.54 | 151.29 | -8.05 | 15.66 | 13.96 | -10.86 | 37.09 | 29.9 | -19.39 | 37.43 | 32.07 | -14.32 |
| **78** | 156.05 | 139.2 | -10.8 | 13.43 | 12.69 | -5.51 | 35.44 | 29.12 | -17.83 | 41.16 | 31.67 | -23.06 |
| **79** | 122.94 | 108.76 | -11.53 | 14.77 | 14 | -5.21 | 33.59 | 28.31 | -15.72 | 35.4 | 25.1 | -29.1 |
| **80** | 119.42 | 106.62 | -10.72 | 14.37 | 13.58 | -5.5 | 27.4 | 21.91 | -20.04 | 31.83 | 22.84 | -28.24 |
| **81** | 151.4 | 135.86 | -10.26 | 13.81 | 12.17 | -11.88 | 30.9 | 25.67 | -16.93 | 38.71 | 26.54 | -31.44 |
| **82** | 140.18 | 124.02 | -11.53 | 13.01 | 11.73 | -9.84 | 29.59 | 22.12 | -25.25 | 43.53 | 34.14 | -21.57 |
| **Total** | 130.51 | 116 | -11.12 | 13.83 | 12.06 | -12.8 | 28.97 | 22.97 | -20.71 | 34.11 | 25.75 | -24.51 |

| Table S4. mean and variability of full filled grain per panicle, yield , 1000-grain weight and kernel length in the studied rice genotypes under drought stress | | | | | | | | | | | | |
| --- | --- | --- | --- | --- | --- | --- | --- | --- | --- | --- | --- | --- |
| **G** | **Full Filled Grain per Panicle** | | | **Yield (kg/ha)** | | | **1000-Grain Weight** | | | **Kernel Length (mm)** | | |
|  | **Normal** | **Stress** | **Variation  (%)** | **Normal** | **Stress** | **Variation  (%)** | **Normal** | **Stress** | **Variation  (%)** | **Normal** | **Stress** | **Variation  (%)** |
| **1** | 132.11 | 93.67 | -29.1 | 5716.67 | 3373.11 | -41 | 35.04 | 30.97 | -11.62 | 6 | 6.43 | 7.17 |
| **2** | 143.67 | 104.11 | -27.54 | 3946.67 | 2309.56 | -41.48 | 24.31 | 20.31 | -16.45 | 6.92 | 6.94 | 0.29 |
| **3** | 110 | 64.56 | -41.31 | 4797.78 | 2793.56 | -41.77 | 18.5 | 17.14 | -7.35 | 5.61 | 5.66 | 0.89 |
| **4** | 142.89 | 111.44 | -22.01 | 4840.33 | 2871.78 | -40.67 | 24.04 | 20.72 | -13.81 | 6.41 | 5.62 | -12.32 |
| **5** | 148.67 | 111.78 | -24.81 | 4871.11 | 3050.22 | -37.38 | 30.2 | 27.29 | -9.64 | 6.22 | 6.39 | 2.73 |
| **6** | 159.33 | 116.89 | -26.64 | 5060 | 2601.33 | -48.59 | 20.27 | 16.97 | -16.28 | 5.81 | 5.79 | -0.34 |
| **7** | 92.33 | 72.11 | -21.9 | 4682.33 | 2501.67 | -46.57 | 22.37 | 17.38 | -22.31 | 6.66 | 6.48 | -2.7 |
| **8** | 107.89 | 73.33 | -32.03 | 3887.67 | 2429.33 | -37.51 | 25.87 | 22.6 | -12.64 | 7.91 | 6.82 | -13.78 |
| **9** | 113.89 | 81.44 | -28.49 | 5210 | 3220 | -38.2 | 31.69 | 28.22 | -10.95 | 6.6 | 6 | -9.09 |
| **10** | 115.11 | 93.67 | -18.63 | 5417.78 | 3654.22 | -32.55 | 34.32 | 33.28 | -3.03 | 5.67 | 5.94 | 4.76 |
| **11** | 136.78 | 88.78 | -35.09 | 5884.67 | 3138.44 | -46.67 | 25.08 | 20.53 | -18.14 | 6.69 | 5.99 | -10.46 |
| **12** | 136 | 123.89 | -8.9 | 4687.78 | 3356.44 | -28.4 | 27.13 | 24.09 | -11.21 | 6.19 | 6.29 | 1.62 |
| **13** | 134.33 | 104.56 | -22.16 | 4816.67 | 3165.78 | -34.27 | 20.69 | 17.6 | -14.93 | 5.74 | 6.32 | 10.1 |
| **14** | 175.56 | 130.67 | -25.57 | 5052.44 | 3228.67 | -36.1 | 25.94 | 22.36 | -13.8 | 6.29 | 6.03 | -4.13 |
| **15** | 78.11 | 57.44 | -26.46 | 3866.67 | 2356.44 | -39.06 | 32.56 | 28.11 | -13.67 | 6.32 | 6.31 | -0.16 |
| **16** | 128.89 | 81.44 | -36.81 | 4017.78 | 2182.89 | -45.67 | 27.63 | 22.06 | -20.16 | 7.92 | 7.09 | -10.48 |
| **17** | 106.67 | 85.78 | -19.58 | 4876 | 2923.78 | -40.04 | 24.92 | 21.34 | -14.37 | 6.77 | 6.48 | -4.28 |
| **18** | 164.22 | 125.44 | -23.61 | 4696.78 | 3175.33 | -32.39 | 26.52 | 22.1 | -16.67 | 6.72 | 6.78 | 0.89 |
| **19** | 87.67 | 67.11 | -23.45 | 4103.33 | 2538 | -38.15 | 29.2 | 25.42 | -12.95 | 7.17 | 7.07 | -1.39 |
| **20** | 107.56 | 79.67 | -25.93 | 4230 | 2676.22 | -36.73 | 22.66 | 20.64 | -8.91 | 5.61 | 5.98 | 6.6 |
| **21** | 101.89 | 75.67 | -25.73 | 3619.44 | 2088.67 | -42.29 | 27.16 | 22.16 | -18.41 | 7.09 | 7.18 | 1.27 |
| **22** | 81.11 | 53 | -34.66 | 5139 | 2836.44 | -44.81 | 25.53 | 24.03 | -5.88 | 7 | 7.31 | 4.43 |
| **23** | 79.44 | 69.78 | -12.16 | 3731.33 | 2829.11 | -24.18 | 25.6 | 21.82 | -14.77 | 8.21 | 7.51 | -8.53 |
| **24** | 108.44 | 79.33 | -26.84 | 4870 | 3105.56 | -36.23 | 23.46 | 20.48 | -12.7 | 7.52 | 6.84 | -9.04 |
| **25** | 121 | 85.89 | -29.02 | 5324.44 | 3137.11 | -41.08 | 27.36 | 23.57 | -13.85 | 6.21 | 6.17 | -0.64 |
| **26** | 104.44 | 71.56 | -31.48 | 3620.22 | 2024 | -44.09 | 31.88 | 28.99 | -9.07 | 6.07 | 7.21 | 18.78 |
| **27** | 110.67 | 82 | -25.91 | 4324.44 | 2849.78 | -34.1 | 38.03 | 35.86 | -5.71 | 6.04 | 6.02 | -0.33 |
| **28** | 88 | 63 | -28.41 | 4058.89 | 2557.56 | -36.99 | 37.59 | 33.83 | -10 | 6.57 | 6.7 | 1.98 |
| **29** | 141 | 109.56 | -22.3 | 2693.33 | 1235.33 | -54.13 | 15.22 | 9.97 | -34.49 | 5.54 | 6.36 | 14.8 |
| **30** | 130.44 | 90.33 | -30.75 | 3424.44 | 1871.33 | -45.35 | 22.93 | 21.51 | -6.19 | 7.91 | 6.91 | -12.64 |
| **31** | 111.67 | 79.89 | -28.46 | 3440 | 1739.33 | -49.44 | 30.34 | 26.69 | -12.03 | 7.31 | 6.54 | -10.53 |
| **32** | 131.22 | 98.56 | -24.89 | 5937.78 | 3723.33 | -37.29 | 30.3 | 23.96 | -20.92 | 5.58 | 5.84 | 4.66 |
| **33** | 215.67 | 136.11 | -36.89 | 3763.33 | 2305 | -38.75 | 25.2 | 19.57 | -22.34 | 7.33 | 6.64 | -9.41 |
| **34** | 55.33 | 39.67 | -28.3 | 3711.11 | 2256.89 | -39.19 | 38.8 | 30.56 | -21.24 | 7.4 | 7.06 | -4.59 |
| **35** | 89 | 65.78 | -26.09 | 3542.22 | 2146.89 | -39.39 | 26.87 | 21.98 | -18.2 | 5.58 | 6.89 | 23.48 |
| **36** | 109.67 | 74.44 | -32.12 | 4814 | 2838.44 | -41.04 | 20.62 | 18.47 | -10.43 | 7.21 | 6.58 | -8.74 |
| **37** | 99.78 | 77.89 | -21.94 | 5654.44 | 3567.11 | -36.91 | 22.89 | 20.39 | -10.92 | 7.13 | 7.27 | 1.96 |
| **38** | 111.78 | 83.44 | -25.35 | 4734.89 | 2870 | -39.39 | 20.09 | 16.89 | -15.93 | 6.33 | 6.5 | 2.69 |
| **39** | 181 | 127.11 | -29.77 | 4557.33 | 3307.33 | -27.43 | 30.99 | 26.42 | -14.75 | 7.24 | 6.43 | -11.19 |
| **40** | 103.89 | 79 | -23.96 | 4316.67 | 2704.22 | -37.35 | 36.61 | 32.78 | -10.46 | 6.31 | 6.47 | 2.54 |
| **41** | 125.22 | 67.67 | -45.96 | 3198 | 1801.56 | -43.67 | 21.88 | 16.96 | -22.49 | 6.2 | 6.73 | 8.55 |
| **42** | 111.22 | 77.11 | -30.67 | 5094.44 | 2921.56 | -42.65 | 33.83 | 29.59 | -12.53 | 6.49 | 6.42 | -1.08 |
| **43** | 114 | 87.22 | -23.49 | 4237.78 | 2914.22 | -31.23 | 36.11 | 32.63 | -9.64 | 6.67 | 6.6 | -1.05 |
| **44** | 172.44 | 129.78 | -24.74 | 5846.67 | 3687.11 | -36.94 | 31.21 | 29.96 | -4.01 | 6.99 | 6.24 | -10.73 |
| **45** | 157.22 | 113.33 | -27.92 | 4810 | 3240.67 | -32.63 | 33.62 | 31.94 | -5 | 6.3 | 6.34 | 0.63 |
| **46** | 146.67 | 109.67 | -25.23 | 5750 | 3395.11 | -40.95 | 38.24 | 36.31 | -5.05 | 7.72 | 6.8 | -11.92 |
| **47** | 49.11 | 39.22 | -20.14 | 1194.44 | 847.33 | -29.06 | 18.18 | 14.43 | -20.63 | 6.99 | 6.67 | -4.58 |
| **48** | 102.89 | 72.67 | -29.37 | 4112.78 | 2543.11 | -38.17 | 24.54 | 21.88 | -10.84 | 6.82 | 6.58 | -3.52 |
| **49** | 113.33 | 82.78 | -26.96 | 4372.22 | 2640.22 | -39.61 | 30.82 | 25.73 | -16.52 | 7.74 | 7.23 | -6.59 |
| **50** | 215.67 | 126.78 | -41.22 | 3104 | 1199.78 | -61.35 | 18.5 | 15.11 | -18.32 | 5.43 | 5.74 | 5.71 |
| **51** | 88.22 | 68.67 | -22.16 | 4756.78 | 2867.11 | -39.73 | 27.12 | 21.5 | -20.72 | 6.82 | 6.86 | 0.59 |
| **52** | 128.33 | 88 | -31.43 | 4865.22 | 2791.33 | -42.63 | 26.58 | 21.22 | -20.17 | 6.86 | 6.18 | -9.91 |
| **53** | 97.44 | 74.78 | -23.26 | 5018.11 | 3031.33 | -39.59 | 26.08 | 22.61 | -13.31 | 7.61 | 7.39 | -2.89 |
| **54** | 126.56 | 90.22 | -28.71 | 4080 | 2377.33 | -41.73 | 28.59 | 27.42 | -4.09 | 7.96 | 7.38 | -7.29 |
| **55** | 135 | 94.67 | -29.87 | 4644.44 | 2722.22 | -41.39 | 24.52 | 22.74 | -7.26 | 8.06 | 7.16 | -11.17 |
| **56** | 94.22 | 82.22 | -12.74 | 5115.56 | 3577.33 | -30.07 | 34.99 | 30.43 | -13.03 | 5.3 | 5.78 | 9.06 |
| **57** | 107.11 | 75 | -29.98 | 3818.89 | 2376.89 | -37.76 | 33.59 | 31.38 | -6.58 | 6.96 | 6.5 | -6.61 |
| **58** | 173.33 | 119.78 | -30.89 | 4167.33 | 2063.44 | -50.49 | 17.01 | 13.76 | -19.11 | 5.62 | 6.08 | 8.19 |
| **59** | 119.56 | 86.22 | -27.89 | 4213.33 | 2487.78 | -40.95 | 26.68 | 23.57 | -11.66 | 6.79 | 6.74 | -0.74 |
| **60** | 105.78 | 80 | -24.37 | 3823.33 | 2308.44 | -39.62 | 27.13 | 25.18 | -7.19 | 5.77 | 6.26 | 8.49 |
| **61** | 125.89 | 79 | -37.25 | 5053.33 | 2718.67 | -46.2 | 28.38 | 23.04 | -18.82 | 7.17 | 6.54 | -8.79 |
| **62** | 102.78 | 73.56 | -28.43 | 3854.44 | 2302.22 | -40.27 | 28.97 | 26.83 | -7.39 | 6.67 | 7.01 | 5.1 |
| **63** | 173.22 | 137.33 | -20.72 | 5364.44 | 3438 | -35.91 | 30.96 | 24.87 | -19.67 | 7.08 | 6.93 | -2.12 |
| **64** | 91.78 | 68.78 | -25.06 | 3898.89 | 2662.22 | -31.72 | 26.32 | 24.21 | -8.02 | 7.33 | 6.81 | -7.09 |
| **65** | 84.89 | 61.89 | -27.09 | 4150 | 2429.11 | -41.47 | 36.46 | 31.06 | -14.81 | 5.96 | 6.02 | 1.01 |
| **66** | 115.11 | 82.78 | -28.09 | 4005.56 | 2285.56 | -42.94 | 24.88 | 20.34 | -18.25 | 7.42 | 6.78 | -8.63 |
| **67** | 117.89 | 83.67 | -29.03 | 3257.78 | 1924.22 | -40.93 | 22.87 | 21.02 | -8.09 | 6.68 | 5.99 | -10.33 |
| **68** | 129.22 | 89.44 | -30.78 | 4128.89 | 2503.78 | -39.36 | 22.68 | 18.23 | -19.62 | 7.24 | 7.03 | -2.9 |
| **69** | 94.44 | 75.56 | -19.99 | 3864 | 2534.89 | -34.4 | 29.53 | 27.42 | -7.15 | 7.77 | 7.39 | -4.89 |
| **70** | 77.33 | 67.78 | -12.35 | 4776.56 | 3251.33 | -31.93 | 28.42 | 24.83 | -12.63 | 7.57 | 7.08 | -6.47 |
| **71** | 92.44 | 72.89 | -21.15 | 4483.33 | 2699.33 | -39.79 | 26.38 | 23.68 | -10.24 | 7.39 | 7.1 | -3.92 |
| **72** | 114.33 | 94.33 | -17.49 | 5365.56 | 2875.56 | -46.41 | 27.92 | 26 | -6.88 | 8.13 | 7.51 | -7.63 |
| **73** | 75.78 | 60.22 | -20.53 | 3592.44 | 2301.56 | -35.93 | 39.07 | 34.57 | -11.52 | 8.27 | 7.11 | -14.03 |
| **74** | 123.22 | 103.67 | -15.87 | 5799.44 | 3528.22 | -39.16 | 26.89 | 24.26 | -9.78 | 7.63 | 6.97 | -8.65 |
| **75** | 96.44 | 74.11 | -23.15 | 3575.33 | 2054.67 | -42.53 | 23.69 | 20.76 | -12.37 | 6.49 | 6.6 | 1.69 |
| **76** | 85 | 61.56 | -27.58 | 3870 | 2236 | -42.22 | 27.99 | 26.73 | -4.5 | 7.09 | 7.13 | 0.56 |
| **77** | 101.89 | 80.56 | -20.93 | 4724.56 | 3146.22 | -33.41 | 29.88 | 23.49 | -21.39 | 6.42 | 6.66 | 3.74 |
| **78** | 89.11 | 64.78 | -27.3 | 4314.89 | 2688.22 | -37.7 | 28.59 | 25.39 | -11.19 | 7.22 | 6.23 | -13.71 |
| **79** | 115.44 | 80.78 | -30.02 | 5599 | 2706.44 | -51.66 | 29.32 | 27.68 | -5.59 | 8.12 | 7.49 | -7.76 |
| **80** | 121.89 | 87 | -28.62 | 5204.44 | 2862.89 | -44.99 | 28.32 | 23.69 | -16.35 | 7.54 | 7.28 | -3.45 |
| **81** | 135.78 | 111.11 | -18.17 | 4140 | 2497.33 | -39.68 | 26.16 | 21.64 | -17.28 | 6.57 | 6.1 | -7.15 |
| **82** | 105.11 | 73.22 | -30.34 | 3934.44 | 2428.44 | -38.28 | 28.87 | 23.04 | -20.19 | 7.69 | 7.38 | -4.03 |
| **Total** | 117.68 | 86.09 | -26.84 | 4428.3 | 2675.72 | -39.58 | 27.66 | 24.1 | -12.87 | 6.82 | 6.63 | -2.79 |

| Table S5. Tolerance and susceptibility index of 82 rice genotypes under conditions of drought stress | | | | | | | | | | | | | | |
| --- | --- | --- | --- | --- | --- | --- | --- | --- | --- | --- | --- | --- | --- | --- |
| Genotype | G | Yn | Ys | Yt | SSI | TOL | MP | GMP | STI | YI | YSI | HM | RDI | ATI |
| A16 | 1 | 5716.67 | 3373.11 | 4544.89 | 1.49 | 2343.56 | 4544.89 | 4391.24 | 136.6 | 1.26 | 0.59 | 4242.78 | 0.98 | 6218215.2 |
| Ak-ypyk | 2 | 3946.67 | 2309.56 | 3128.11 | 1.48 | 1637.11 | 3128.11 | 3019.11 | 94.01 | 0.86 | 0.59 | 2913.91 | 0.97 | 2986491.8 |
| Amber albalaka | 3 | 4797.78 | 2793.56 | 3795.67 | 1.47 | 2004.22 | 3795.67 | 3660.99 | 114.08 | 1.04 | 0.58 | 3531.09 | 0.96 | 4433516.5 |
| Anbar33 | 4 | 4840.33 | 2871.78 | 3856.06 | 1.5 | 1968.56 | 3856.06 | 3728.32 | 115.89 | 1.07 | 0.59 | 3604.81 | 0.98 | 4434702.2 |
| Atai-1 | 5 | 4871.11 | 3050.22 | 3960.67 | 1.58 | 1820.89 | 3960.67 | 3854.6 | 119.04 | 1.14 | 0.63 | 3751.38 | 1.04 | 4240987 |
| Barnamaj4 | 6 | 5060 | 2601.33 | 3830.67 | 1.3 | 2458.67 | 3830.67 | 3628.05 | 115.13 | 0.97 | 0.51 | 3436.15 | 0.85 | 5389849.7 |
| BT7 | 7 | 4682.33 | 2501.67 | 3592 | 1.35 | 2180.67 | 3592 | 3422.52 | 107.96 | 0.93 | 0.53 | 3261.03 | 0.88 | 4509609.2 |
| C10 | 8 | 3887.67 | 2429.33 | 3158.5 | 1.58 | 1458.33 | 3158.5 | 3073.18 | 94.93 | 0.91 | 0.62 | 2990.17 | 1.03 | 2707999.7 |
| Cakmak | 9 | 5210 | 3220 | 4215 | 1.56 | 1990 | 4215 | 4095.88 | 126.68 | 1.2 | 0.62 | 3980.12 | 1.02 | 4924970.2 |
| D3 | 10 | 5417.78 | 3654.22 | 4536 | 1.7 | 1763.56 | 4536 | 4449.47 | 136.33 | 1.37 | 0.67 | 4364.59 | 1.12 | 4741338.4 |
| Dijla | 11 | 5884.67 | 3138.44 | 4511.56 | 1.35 | 2746.22 | 4511.56 | 4297.52 | 135.59 | 1.17 | 0.53 | 4093.64 | 0.88 | 7131117.1 |
| Dollar | 12 | 4687.78 | 3356.44 | 4022.11 | 1.81 | 1331.33 | 4022.11 | 3966.64 | 120.88 | 1.25 | 0.72 | 3911.94 | 1.18 | 3190904.3 |
| Furat1 | 13 | 4816.67 | 3165.78 | 3991.22 | 1.66 | 1650.89 | 3991.22 | 3904.93 | 119.95 | 1.18 | 0.66 | 3820.51 | 1.09 | 3895247.8 |
| Ghadeer | 14 | 5052.44 | 3228.67 | 4140.56 | 1.61 | 1823.78 | 4140.56 | 4038.89 | 124.44 | 1.21 | 0.64 | 3939.73 | 1.06 | 4450799.5 |
| Halibey | 15 | 3866.67 | 2356.44 | 3111.56 | 1.54 | 1510.22 | 3111.56 | 3018.54 | 93.52 | 0.88 | 0.61 | 2928.31 | 1.01 | 2754492.3 |
| Hikkan Hashimi | 16 | 4017.78 | 2182.89 | 3100.33 | 1.37 | 1834.89 | 3100.33 | 2961.48 | 93.18 | 0.82 | 0.54 | 2828.85 | 0.9 | 3283388.6 |
| HT1 | 17 | 4876 | 2923.78 | 3899.89 | 1.52 | 1952.22 | 3899.89 | 3775.76 | 117.21 | 1.09 | 0.6 | 3655.58 | 0.99 | 4453864.7 |
| Iba | 18 | 4696.78 | 3175.33 | 3936.06 | 1.71 | 1521.44 | 3936.06 | 3861.84 | 118.3 | 1.19 | 0.68 | 3789.03 | 1.12 | 3550213.8 |
| IR28 | 19 | 4103.33 | 2538 | 3320.67 | 1.56 | 1565.33 | 3320.67 | 3227.11 | 99.8 | 0.95 | 0.62 | 3136.2 | 1.02 | 3052282.9 |
| IR30 | 20 | 4230 | 2676.22 | 3453.11 | 1.6 | 1553.78 | 3453.11 | 3364.58 | 103.78 | 1 | 0.63 | 3278.32 | 1.05 | 3158813 |
| IR36 | 21 | 3619.44 | 2088.67 | 2854.06 | 1.46 | 1530.78 | 2854.06 | 2749.51 | 85.78 | 0.78 | 0.58 | 2648.8 | 0.96 | 2543146.7 |
| IR50 | 22 | 5139 | 2836.44 | 3987.72 | 1.39 | 2302.56 | 3987.72 | 3817.92 | 119.85 | 1.06 | 0.55 | 3655.34 | 0.91 | 5311782.8 |
| IR58 | 23 | 3731.33 | 2829.11 | 3280.22 | 1.92 | 902.22 | 3280.22 | 3249.05 | 98.59 | 1.06 | 0.76 | 3218.18 | 1.25 | 1771227.2 |
| IR60 | 24 | 4870 | 3105.56 | 3987.78 | 1.61 | 1764.44 | 3987.78 | 3888.97 | 119.85 | 1.16 | 0.64 | 3792.6 | 1.06 | 4146158.1 |
| IR64 | 25 | 5324.44 | 3137.11 | 4230.78 | 1.49 | 2187.33 | 4230.78 | 4086.98 | 127.15 | 1.17 | 0.59 | 3948.06 | 0.98 | 5401580 |
| Iskander | 26 | 3620.22 | 2024 | 2822.11 | 1.41 | 1596.22 | 2822.11 | 2706.9 | 84.82 | 0.76 | 0.56 | 2596.4 | 0.93 | 2610778.1 |
| Jalal Abad | 27 | 4324.44 | 2849.78 | 3587.11 | 1.67 | 1474.67 | 3587.11 | 3510.51 | 107.81 | 1.07 | 0.66 | 3435.55 | 1.09 | 3128010.9 |
| Kapa | 28 | 4058.89 | 2557.56 | 3308.22 | 1.59 | 1501.33 | 3308.22 | 3221.93 | 99.43 | 0.96 | 0.63 | 3137.89 | 1.04 | 2922785.8 |
| Kawther | 29 | 2693.33 | 1235.33 | 1964.33 | 1.16 | 1458 | 1964.33 | 1824.05 | 59.04 | 0.46 | 0.46 | 1693.79 | 0.76 | 1606935.3 |
| Labypma | 30 | 3424.44 | 1871.33 | 2647.89 | 1.38 | 1553.11 | 2647.89 | 2531.46 | 79.58 | 0.7 | 0.55 | 2420.15 | 0.9 | 2375619.6 |
| LT2 | 31 | 3440 | 1739.33 | 2589.67 | 1.28 | 1700.67 | 2589.67 | 2446.08 | 77.83 | 0.65 | 0.51 | 2310.45 | 0.84 | 2513585 |
| M1 | 32 | 5937.78 | 3723.33 | 4830.56 | 1.58 | 2214.44 | 4830.56 | 4701.95 | 145.18 | 1.39 | 0.63 | 4576.77 | 1.04 | 6291387.8 |
| Manyas Yildizi | 33 | 3763.33 | 2305 | 3034.17 | 1.55 | 1458.33 | 3034.17 | 2945.25 | 91.19 | 0.86 | 0.61 | 2858.93 | 1.01 | 2595268.8 |
| Marjan | 34 | 3711.11 | 2256.89 | 2984 | 1.54 | 1454.22 | 2984 | 2894.06 | 89.68 | 0.84 | 0.61 | 2806.82 | 1.01 | 2542972 |
| Mis-2013 | 35 | 3542.22 | 2146.89 | 2844.56 | 1.53 | 1395.33 | 2844.56 | 2757.67 | 85.49 | 0.8 | 0.61 | 2673.44 | 1 | 2325007.7 |
| Mishkab1 | 36 | 4814 | 2838.44 | 3826.22 | 1.49 | 1975.56 | 3826.22 | 3696.52 | 115 | 1.06 | 0.59 | 3571.22 | 0.98 | 4412515.3 |
| Mishkab2 | 37 | 5654.44 | 3567.11 | 4610.78 | 1.59 | 2087.33 | 4610.78 | 4491.11 | 138.58 | 1.33 | 0.63 | 4374.54 | 1.04 | 5664333.8 |
| Mustakillik | 38 | 4734.89 | 2870 | 3802.44 | 1.53 | 1864.89 | 3802.44 | 3686.34 | 114.28 | 1.07 | 0.61 | 3573.79 | 1 | 4153866.6 |
| Okean | 39 | 4557.33 | 3307.33 | 3932.33 | 1.83 | 1250 | 3932.33 | 3882.35 | 118.18 | 1.24 | 0.73 | 3833 | 1.2 | 2932298.2 |
| osmancik-97 | 40 | 4316.67 | 2704.22 | 3510.44 | 1.58 | 1612.44 | 3510.44 | 3416.61 | 105.51 | 1.01 | 0.63 | 3325.28 | 1.04 | 3328771.2 |
| Avangard | 41 | 3198 | 1801.56 | 2499.78 | 1.42 | 1396.44 | 2499.78 | 2400.29 | 75.13 | 0.67 | 0.56 | 2304.75 | 0.93 | 2025305.1 |
| Pasali | 42 | 5094.44 | 2921.56 | 4008 | 1.45 | 2172.89 | 4008 | 3857.94 | 120.46 | 1.09 | 0.57 | 3713.5 | 0.95 | 5065201.7 |
| QazNIIR-7 | 43 | 4237.78 | 2914.22 | 3576 | 1.74 | 1323.56 | 3576 | 3514.23 | 107.48 | 1.09 | 0.69 | 3453.53 | 1.14 | 2810453.1 |
| Sela-Zodras | 44 | 5846.67 | 3687.11 | 4766.89 | 1.59 | 2159.56 | 4766.89 | 4642.98 | 143.27 | 1.38 | 0.63 | 4522.3 | 1.04 | 6058504 |
| Shalawangi 1 | 45 | 4810 | 3240.67 | 4025.33 | 1.7 | 1569.33 | 4025.33 | 3948.11 | 120.98 | 1.21 | 0.67 | 3872.38 | 1.12 | 3743765.7 |
| Shalawangi 2 | 46 | 5750 | 3395.11 | 4572.56 | 1.49 | 2354.89 | 4572.56 | 4418.36 | 137.43 | 1.27 | 0.59 | 4269.36 | 0.98 | 6286878.6 |
| Siyavar Hasimi | 47 | 1194.44 | 847.33 | 1020.89 | 1.79 | 347.11 | 1020.89 | 1006.03 | 30.68 | 0.32 | 0.71 | 991.38 | 1.17 | 210999.96 |
| Sumer | 48 | 4112.78 | 2543.11 | 3327.94 | 1.56 | 1569.67 | 3327.94 | 3234.08 | 100.02 | 0.95 | 0.62 | 3142.86 | 1.02 | 3067336.8 |
| Syl Sulu | 49 | 4372.22 | 2640.22 | 3506.22 | 1.53 | 1732 | 3506.22 | 3397.59 | 105.38 | 0.99 | 0.6 | 3292.33 | 1 | 3555682.5 |
| T85 | 50 | 3104 | 1199.78 | 2151.89 | 0.98 | 1904.22 | 2151.89 | 1929.8 | 64.67 | 0.45 | 0.39 | 1730.62 | 0.64 | 2220407.2 |
| Tantana | 51 | 4756.78 | 2867.11 | 3811.94 | 1.52 | 1889.67 | 3811.94 | 3692.99 | 114.57 | 1.07 | 0.6 | 3577.76 | 1 | 4216650.8 |
| Tarona | 52 | 4865.22 | 2791.33 | 3828.28 | 1.45 | 2073.89 | 3828.28 | 3685.17 | 115.06 | 1.04 | 0.57 | 3547.41 | 0.95 | 4617920.1 |
| TosyaGunesi | 53 | 5018.11 | 3031.33 | 4024.72 | 1.53 | 1986.78 | 4024.72 | 3900.2 | 120.96 | 1.13 | 0.6 | 3779.53 | 1 | 4682092.8 |
| V20-48(awn) | 54 | 4080 | 2377.33 | 3228.67 | 1.47 | 1702.67 | 3228.67 | 3114.41 | 97.04 | 0.89 | 0.58 | 3004.19 | 0.96 | 3204117.9 |
| V20-53-2-2 | 55 | 4644.44 | 2722.22 | 3683.33 | 1.48 | 1922.22 | 3683.33 | 3555.73 | 110.7 | 1.02 | 0.59 | 3432.55 | 0.97 | 4129866.4 |
| V20-8-2 | 56 | 5115.56 | 3577.33 | 4346.44 | 1.77 | 1538.22 | 4346.44 | 4277.86 | 130.63 | 1.34 | 0.7 | 4210.35 | 1.16 | 3976023.3 |
| Xazaz Hazar | 57 | 3818.89 | 2376.89 | 3097.89 | 1.57 | 1442 | 3097.89 | 3012.82 | 93.11 | 0.89 | 0.62 | 2930.08 | 1.03 | 2625076.4 |
| Yasmine | 58 | 4167.33 | 2063.44 | 3115.39 | 1.25 | 2103.89 | 3115.39 | 2932.42 | 93.63 | 0.77 | 0.5 | 2760.19 | 0.82 | 3727795.2 |
| Ahlami Tarom | 59 | 4213.33 | 2487.78 | 3350.56 | 1.49 | 1725.56 | 3350.56 | 3237.57 | 100.7 | 0.93 | 0.59 | 3128.39 | 0.98 | 3375603.2 |
| Abjiboji | 60 | 3823.33 | 2308.44 | 3065.89 | 1.53 | 1514.89 | 3065.89 | 2970.85 | 92.14 | 0.86 | 0.6 | 2878.76 | 1 | 2719351.3 |
| Bojar | 61 | 5053.33 | 2718.67 | 3886 | 1.36 | 2334.67 | 3886 | 3706.53 | 116.79 | 1.02 | 0.54 | 3535.34 | 0.89 | 5228722.3 |
| Binam | 62 | 3854.44 | 2302.22 | 3078.33 | 1.51 | 1552.22 | 3078.33 | 2978.89 | 92.52 | 0.86 | 0.6 | 2882.66 | 0.99 | 2793908.3 |
| Champa Boodar | 63 | 5364.44 | 3438 | 4401.22 | 1.62 | 1926.44 | 4401.22 | 4294.53 | 132.28 | 1.28 | 0.64 | 4190.42 | 1.06 | 4998912.5 |
| Hasansaraei | 64 | 3898.89 | 2662.22 | 3280.56 | 1.73 | 1236.67 | 3280.56 | 3221.76 | 98.6 | 0.99 | 0.68 | 3164.01 | 1.13 | 2407404 |
| Hasani | 65 | 4150 | 2429.11 | 3289.56 | 1.48 | 1720.89 | 3289.56 | 3175.03 | 98.87 | 0.91 | 0.59 | 3064.49 | 0.97 | 3301446.8 |
| Khazar | 66 | 4005.56 | 2285.56 | 3145.56 | 1.44 | 1720 | 3145.56 | 3025.71 | 94.54 | 0.85 | 0.57 | 2910.43 | 0.94 | 3144556.9 |
| Domzard | 67 | 3257.78 | 1924.22 | 2591 | 1.49 | 1333.56 | 2591 | 2503.73 | 77.87 | 0.72 | 0.59 | 2419.41 | 0.98 | 2017451.9 |
| Domsefid | 68 | 4128.89 | 2503.78 | 3316.33 | 1.53 | 1625.11 | 3316.33 | 3215.25 | 99.67 | 0.94 | 0.61 | 3117.24 | 1 | 3157194.1 |
| Domsiah | 69 | 3864 | 2534.89 | 3199.44 | 1.66 | 1329.11 | 3199.44 | 3129.67 | 96.16 | 0.95 | 0.66 | 3061.41 | 1.09 | 2513408.3 |
| Sahel | 70 | 4776.56 | 3251.33 | 4013.94 | 1.72 | 1525.22 | 4013.94 | 3940.83 | 120.64 | 1.22 | 0.68 | 3869.06 | 1.13 | 3631825.9 |
| Salari | 71 | 4483.33 | 2699.33 | 3591.33 | 1.52 | 1784 | 3591.33 | 3478.79 | 107.94 | 1.01 | 0.6 | 3369.78 | 1 | 3749966.3 |
| SangTarom | 72 | 5365.56 | 2875.56 | 4120.56 | 1.35 | 2490 | 4120.56 | 3927.97 | 123.84 | 1.07 | 0.54 | 3744.39 | 0.89 | 5909780 |
| Shahpasand | 73 | 3592.44 | 2301.56 | 2947 | 1.62 | 1290.89 | 2947 | 2875.45 | 88.57 | 0.86 | 0.64 | 2805.64 | 1.06 | 2242840.4 |
| Shiroudi | 74 | 5799.44 | 3528.22 | 4663.83 | 1.54 | 2271.22 | 4663.83 | 4523.46 | 140.17 | 1.32 | 0.61 | 4387.32 | 1.01 | 6207754.4 |
| Saleh | 75 | 3575.33 | 2054.67 | 2815 | 1.45 | 1520.67 | 2815 | 2710.37 | 84.6 | 0.77 | 0.57 | 2609.63 | 0.95 | 2490386.3 |
| Alikazemi | 76 | 3870 | 2236 | 3053 | 1.46 | 1634 | 3053 | 2941.65 | 91.76 | 0.84 | 0.58 | 2834.37 | 0.96 | 2904338.1 |
| Anbarboo | 77 | 4724.56 | 3146.22 | 3935.39 | 1.68 | 1578.33 | 3935.39 | 3855.45 | 118.28 | 1.18 | 0.67 | 3777.14 | 1.1 | 3676864.6 |
| Gharib | 78 | 4314.89 | 2688.22 | 3501.56 | 1.57 | 1626.67 | 3501.56 | 3405.79 | 105.24 | 1 | 0.62 | 3312.64 | 1.03 | 3347493.1 |
| Kadous | 79 | 5599 | 2706.44 | 4152.72 | 1.22 | 2892.56 | 4152.72 | 3892.73 | 124.81 | 1.01 | 0.48 | 3649.02 | 0.8 | 6803623 |
| Gilaneh | 80 | 5204.44 | 2862.89 | 4033.67 | 1.39 | 2341.56 | 4033.67 | 3860.02 | 121.23 | 1.07 | 0.55 | 3693.85 | 0.91 | 5461320.2 |
| Mohammadi | 81 | 4140 | 2497.33 | 3318.67 | 1.52 | 1642.67 | 3318.67 | 3215.43 | 99.74 | 0.93 | 0.6 | 3115.4 | 1 | 3191476.2 |
| Hashemi | 82 | 3934.44 | 2428.44 | 3181.44 | 1.56 | 1506 | 3181.44 | 3091.05 | 95.62 | 0.91 | 0.62 | 3003.22 | 1.02 | 2812771.8 |

| 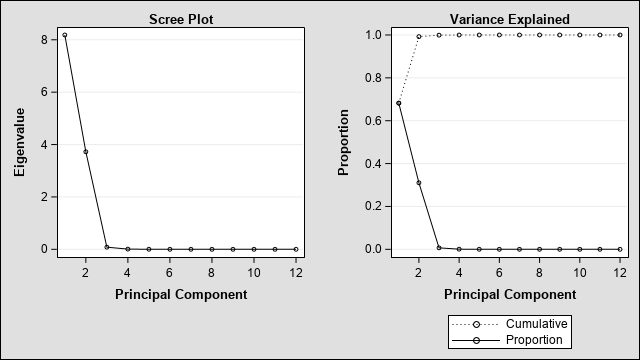 |
| --- |
| Figure S1. Scree plot and variance explained of Principal Component Analysis (PCA) |
